# Genetic evidence for the causal relationships between migraine, dementia, and longitudinal brain atrophy

**DOI:** 10.1186/s10194-024-01801-7

**Published:** 2024-06-05

**Authors:** Lei Zhao, Yilan Tang, Yiheng Tu, Jin Cao

**Affiliations:** 1 CAS Key Laboratory of Mental Health, Institute of Psychology, Chinese Academy of Sciences, 16 Lincui Road, Beijing, China; 2 Department of Psychology, University of Chinese Academy of Sciences, 16 Lincui Road, Beijing, China; 3 School of Life Sciences, Beijing University of Chinese Medicine, 11 North third Ring Road East, Beijing, China

**Keywords:** Dementia, Migraine, Longitudinal brain measures, Mendelian randomization, Brain atrophy, Causal associations

## Abstract

**Background:**

Migraine is a neurological disease with a significant genetic component and is characterized by recurrent and prolonged episodes of headache. Previous epidemiological studies have reported a higher risk of dementia in migraine patients. Neuroimaging studies have also shown structural brain atrophy in regions that are common to migraine and dementia. However, these studies are observational and cannot establish causality. The present study aims to explore the genetic causal relationship between migraine and dementia, as well as the mediation roles of brain structural changes in this association using Mendelian randomization (MR).

**Methods:**

We collected the genome-wide association study (GWAS) summary statistics of migraine and its two subtypes, as well as four common types of dementia, including Alzheimer’s disease (AD), vascular dementia, frontotemporal dementia, and Lewy body dementia. In addition, we collected the GWAS summary statistics of seven longitudinal brain measures that characterize brain structural alterations with age. Using these GWAS, we performed Two-sample MR analyses to investigate the causal effects of migraine and its two subtypes on dementia and brain structural changes. To explore the possible mediation of brain structural changes between migraine and dementia, we conducted a two-step MR mediation analysis.

**Results:**

The MR analysis demonstrated a significant association between genetically predicted migraine and an increased risk of AD (OR = 1.097, 95% CI = [1.040, 1.158], *p* = 7.03 × 10^− 4^). Moreover, migraine significantly accelerated annual atrophy of the total cortical surface area (-65.588 cm^2^ per year, 95% CI = [-103.112, -28.064], *p* = 6.13 × 10^− 4^) and thalamic volume (-9.507 cm^3^ per year, 95% CI = [-15.512, -3.502], *p* = 1.91 × 10^− 3^). The migraine without aura (MO) subtype increased the risk of AD (OR = 1.091, 95% CI = [1.059, 1.123], *p* = 6.95 × 10^− 9^) and accelerated annual atrophy of the total cortical surface area (-31.401 cm^2^ per year, 95% CI = [-43.990, -18.811], *p* = 1.02 × 10^− 6^). The two-step MR mediation analysis revealed that thalamic atrophy partly mediated the causal effect of migraine on AD, accounting for 28.2% of the total effect.

**Discussion:**

This comprehensive MR study provided genetic evidence for the causal effect of migraine on AD and identified longitudinal thalamic atrophy as a potential mediator in this association. These findings may inform brain intervention targets to prevent AD risk in migraine patients.

**Supplementary Information:**

The online version contains supplementary material available at 10.1186/s10194-024-01801-7.

## Introduction

Migraine is a heritable neurological disease characterized by frequent headache attacks, affecting approximately 15% of the global population [1]. Although migraine is more prevalent in young and middle-aged adults and peaks between 40 and 44 years, then declines with age [2], the neuropathological impairments associated with migraine may persist and increase the risk of dementia later in life. The relationship between migraine and dementia has gained much attention in recent years. Some prospective epidemiologic studies [3–6] have suggested that individuals with a history of migraine are more likely to develop dementia. Migraine subtypes may also differ in their dementia risk, with migraine with aura (MA) having a higher risk than migraine without aura (MO) [5–6].

Neuroimaging studies have provided additional evidence for the link between migraine and dementia by detecting structural brain atrophy of migraine patients in regions that are also affected by dementia, such as the cerebral cortex, thalamus, hippocampus, and caudate [7–14]. In addition, migraine may lead to accelerated brain atrophy [15], which has also been observed in dementia [16–17]. Some longitudinal studies have shown that accelerated brain atrophy can precede the onset of dementia, [18, 19] suggesting a possible mediating role of accelerated brain atrophy in the association between migraine and the development of dementia in the future. However, there are still some inconsistent conclusions between previous observational studies [3–6, 20–22]. The causal relationship between migraine and dementia, as well as the involvement of brain changes in this association, remains unclear due to the potential confounding factors that may influence both conditions in observational studies, such as comorbidity and medical conditions.

To examine the causal effects of migraine and its subtypes on dementia, we used Mendelian randomization (MR), a statistical framework that uses genetic variants of exposures (diseases or risk factors) as instrumental variables (IVs) to investigate their effects on outcomes (diseases or brain structures) [23]. MR can minimize confounding issues in observational data by using genetic variants that are independent of self-selected behaviors and established well before disease onset [24, 25]. Thus, MR can be used to mimic the design of randomized controlled trials. This statistical framework has found widespread application in deducing causal associations between diseases and brain measures and demonstrated its clinical significance [26, 27]. In this study, we first investigated the causal effects of migraine on four common types of dementia using two-sample MR analyses. Leveraging the recent genome-wide association study (GWAS) summary statistics of longitudinal brain measures, which revealed the genetic influences on brain structural alterations with age using magnetic resonance imaging (MRI) data [28], we then investigated the causal effects of migraine on longitudinal brain changes. Furthermore, we replicated the MR estimates in two migraine subtypes (i.e., MA and MO subtypes). Finally, we examined the mediation effects of longitudinal brain changes on the migraine-dementia relationship using a two-step MR mediation analysis.

## Methods

### Two-sample MR study design

**Fig. 1:**
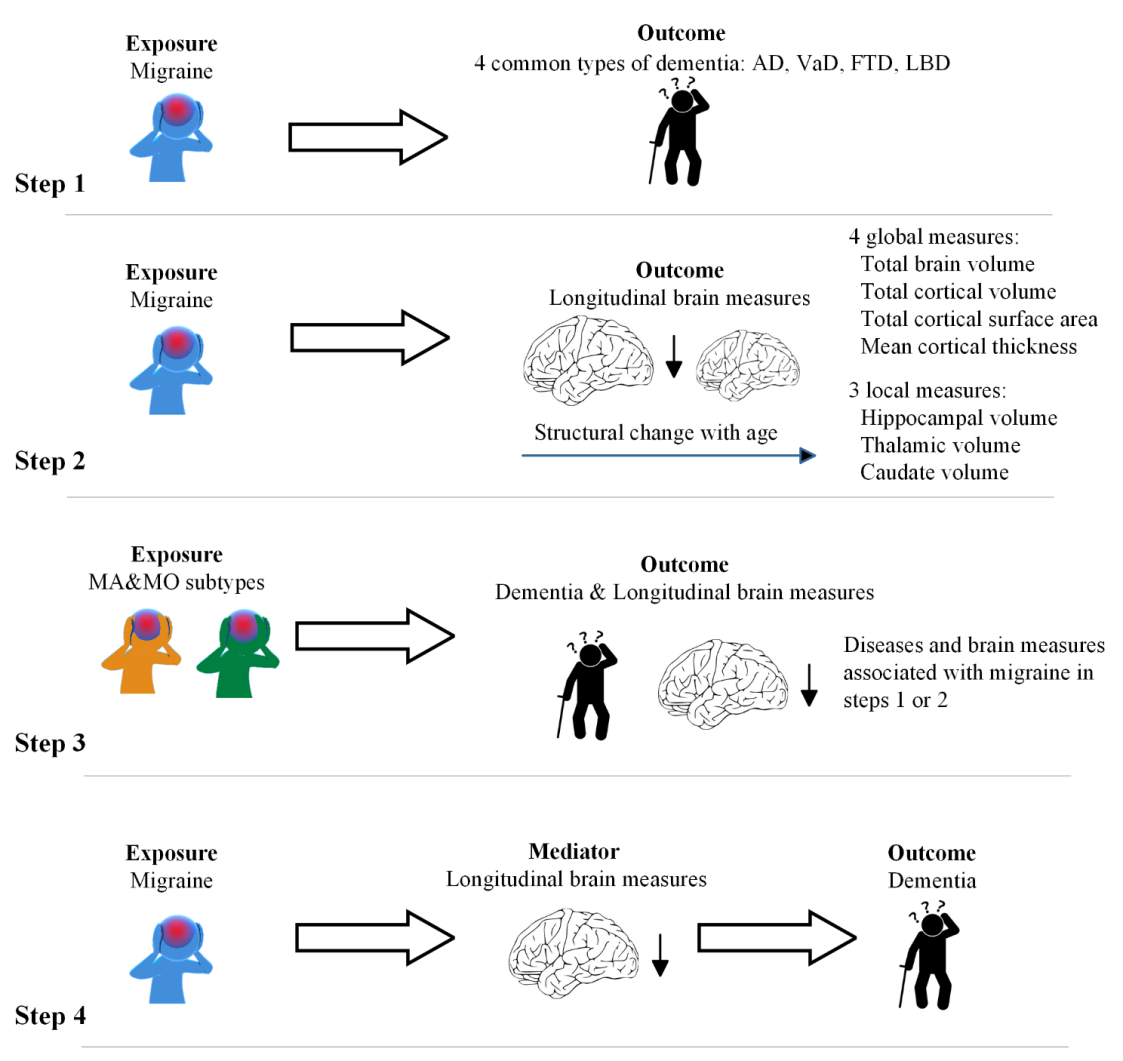
Study design for identification of the causal relationships between migraine, dementia, and longitudinal brain measures. *Abbreviations* AD, Alzheimer’s disease; VaD, vascular dementia; FTD, frontotemporal dementia; LBD, Lewy body dementia; MA, migraine with aura; MO, migraine without aura

We employed Two-sample MR analysis to investigate the genetic causal effects of exposures on outcomes based on GWAS summary statistics. The study design is shown in Fig. 1. In step 1, we assessed the causal effects of migraine on four common types of dementia, including Alzheimer’s disease (AD), vascular dementia (VaD), frontotemporal dementia (FTD), and Lewy body dementia (LBD). In step 2, we examined the causal effects of migraine on longitudinal changes in four global and three local brain measures. In step 3, we conducted a subtype analysis to replicate the findings of steps 1–2. In step 4, we investigated the mediation effects of longitudinal brain measures between migraine and dementia by conducting a two-step MR mediation analysis.

### GWAS of exposures

To avoid the bias resulting from sample overlaps between exposures and outcomes (i.e., samples from the UK biobank) in Two-sample MR analysis, we did not use the latest GWAS summary statistics of migraine [29]. Instead, we included an earlier version of GWAS summary statistics for migraine, which has no sample overlaps with the outcomes used in this study [30]. This GWAS yielded also the genetic variants related to two subtypes of migraine, MA and MO. To ensure privacy protection for participants in the 23andMe cohort, we excluded their samples from the GWAS summary statistics. Consequently, the GWAS summary statistics used in the present study comprised data from 202,140 (Migraine: Ncase = 29,209, Ncontrol = 172,931), 151,215 (MA: Ncase = 6,332, Ncontrol = 144,883), and 147,970 (MO: Ncase = 8,348, Ncontrol = 139,622) individuals, respectively. These participants were recruited from six tertiary headache clinics (*N* = 20,395) and fifteen population-based cohorts (*N* = 181,745) through various methods, such as advertisements, the project’s website, national media campaigns, and referrals from headache centers. Detailed recruitment information is available in the respective cohort descriptions. Migraine cases, including those with current episodes and a history of migraine, were identified using self-reports, diagnostic questionnaires meeting full or modified ICHD-II criteria, or diagnoses by trained physicians or senior medical students. This enabled the inclusion of a large number of cases, thereby enhancing the statistical power. Only individuals who met the strict classification criteria standardized by the International Headache Society were included as migraine subtype cases because the migraine aura can be difficult to distinguish in a questionnaire-based setting.

### GWAS of outcomes

We collected the latest GWAS summary statistics of four common types of dementia, including AD (Ncase = 111,326, Ncontrol = 677,663) [31], VaD (obtained from the FinnGen database R9; Ncase = 2,335, Ncontrol = 360,778), FTD (Ncase = 2,154, Ncontrol = 4,308) [32], and LBD (Ncase = 2,591, Ncontrol = 4,027) [33]. In the AD GWAS, cases were identified using multiple approaches, including clinical diagnosis by experts, self-report of the diagnosis, or self-report of a family history of AD. The inclusion of some proxy cases in the AD GWAS may lower the specificity of the findings. We thus conducted a replication analysis using the AD GWAS in the FinnGen database (R9; Ncase = 9,301, Ncontrol = 367,976) that has an overlap with the AD GWAS used in the primary analysis but only included clinically diagnosed cases. The VaD cases were collected from nationwide electronic health registers in Finland using the International Classification of Diseases, 10th Revision (ICD-10) codes, as defined by FinnGen clinical expert groups. Diagnoses were based on hospital billing codes rather than specific viral assays. The diagnosis of FTD was made by a neurologist using the Neary criteria (97% of the total sample) or in a minority (3% of the total sample), by pathological diagnosis (e.g., TDP-43 and FUS). LBD cases were confirmed based on either pathologically definite criteria (69% of the total sample) or clinically probable criteria (31% of the total sample), as recommended by the Dementia with Lewy Bodies Consortium. The diagnostic process integrated clinical features and biomarkers obtained from imaging and cerebrospinal fluid analyses.

We collected the GWAS of longitudinal brain measures from a study conducted on 15,100 participants of European ancestry where each participant underwent both baseline and follow-up MRI scans [28]. Participants in this study were recruited from various population-based, case-control, and family-based cohorts through multiple methods, including invitation letters, citizen registries, and the project’s website. Detailed recruitment information can be found in the descriptions of the respective cohorts. Considering that genetic risk for disease may be associated with genetic influences on brain changes, both healthy participants (89% of the total sample) and patients with neuropathic or psychiatric disorders (11% of the total sample) were included in the analysis, enhancing the applicability for inferring pathology and the adverse consequences of diseases. This study processed the MRI data using the FreeSurfer, a widely used tool for automated brain morphometry analysis. The phenotypes in this study are annual rates of change of 15 brain imaging-derived measures that are calculated by subtracting baseline brain measures from follow-up measures and dividing by the number of years of follow-up duration. Of the 15 longitudinal brain measures, we included four global (total brain volume, total cortical volume, total cortical surface area, and mean cortical thickness) and three local (volumes of the hippocampus, thalamus, and caudate) brain measures that were strongly associated with aging and presented an almost linear trajectory of change over time. It is noted that all GWAS included in the present study exclusively comprise participants of European ancestry.

### Selection and harmonization of genetic IVs

Before conducting Two-sample MR analyses, we first selected and preprocessed the genetic IVs. The genetic variants with minor allele frequency (MAF) < 1% were removed from GWAS summary statistics. To meet strong associations between IVs and exposures, we selected the genetic IVs with a genome-wide significance threshold of *p* < 5 × 10^− 8^ and F value (β^2^ / se^2^) > 10. When the number of genetic variants reaching the genome-wide significance threshold was no more than 3, we relaxed the significance threshold to *p* < 5 × 10^− 6^ [34, 35]. The resulting genetic IVs were pruned to high independence with a *r*^2^ threshold of 0.001 and a window size of 1 Mb. To ensure that the genetic IVs are associated with outcomes only through exposures, we removed the genetic IVs that were strongly associated with the outcome (*p* < 5 × 10^− 8^). We also removed the Single Nucleotide Polymorphisms (SNPs) located in long LD regions due to their high potential for pleiotropy [36]. After converting all odds ratio (OR) values in case/control GWAS to log odds, the effects of genetic IVs on exposure and outcome were harmonized to the same alleles. Because palindromic SNPs are sensitive to strand-flipping issues that impede the harmonization of effect alleles, we removed the palindromic SNPs (i.e., A/T or G/C alleles) with MAF close to 50%. The genetic IVs that were not available in outcome GWAS were replaced with proxy SNPs (*r*^2^ > 0.8) using a web-based tool “LDlinkR” [37]. The outliers were detected and excluded using the “ivw_radial” (alpha = 0.05, weights = 1, tol = 0.0001) and “egger_radial” (alpha = 0.05, weights = 1) of the “RadialMR” package [38]. 

### Statistical analysis

We used the TwoSampleMR R package (https://mrcieu.github.io/TwoSampleMR) to perform Two-sample MR analyses with the multiplicative random-effects inverse-variance weighted (IVW) estimate as the primary analysis method to evaluate the causal effects of exposures on outcomes. To examine the robustness of the IVW estimate, we employed three supplementary MR methods (weighted median, weighted mode, and MR-Egger method) to conduct MR analysis. The significant threshold was set as two-tailed *p* < 0.05 and corrected for multiple testing with Bonferroni within each step (step 1: 0.05/4 = 0.0125; step 2:0.05/7 = 0.0071; step 3: 0.05/6 = 0.0083).

### Sensitivity analysis

To exclude the potential influence of pleiotropy, we validated our findings by conducting a succession of sensitivity analyses as follows: (1) MR-Egger regression and MR-PRESSO Global test; (2) Cochran’s Q heterogeneity test; (3) leave-one-out (LOO) analysis. We also conducted a replication analysis by excluding potential pleiotropic IVs that were strongly associated with some confounders in European ancestry. Briefly, we searched for SNPs exhibiting significant associations with smoking status, alcohol consumption, major depressive disorder, coronary artery disease, stroke, and hypertension in Phenoscanner (http://www.phenoscanner.medschl.cam.ac.uk) and removed them from the IVs. For the dementia diseases that were significantly affected by migraine, we additionally conducted reversed MR analyses to assess their causal effects on migraine.

### Mediation analysis

We conducted a two-step MR mediation analysis to investigate the mediating pathway from migraine to dementia via longitudinal brain measures. In the first step, we estimated the causal effect of migraine on longitudinal brain measures. In the second step, we assessed the causal effect of longitudinal brain measures on dementia. Finally, we quantified the indirect effect of migraine on dementia via longitudinal brain measures. The “product of coefficients” and “delta” methods were used to assess the indirect effects and their standard errors, respectively [39]. A sample overlap was found between the GWAS of longitudinal brain measures and AD (i.e., samples from the UK biobank). To avoid the bias caused by the sample overlaps in Two-sample MR analysis, we used the GWAS of AD in the FinnGen database (R9; Ncase = 9,301, Ncontrol = 367,976) to complete the mediation analysis.

## Results

### The putative causal effects of migraine on dementia

**Fig. 2:**
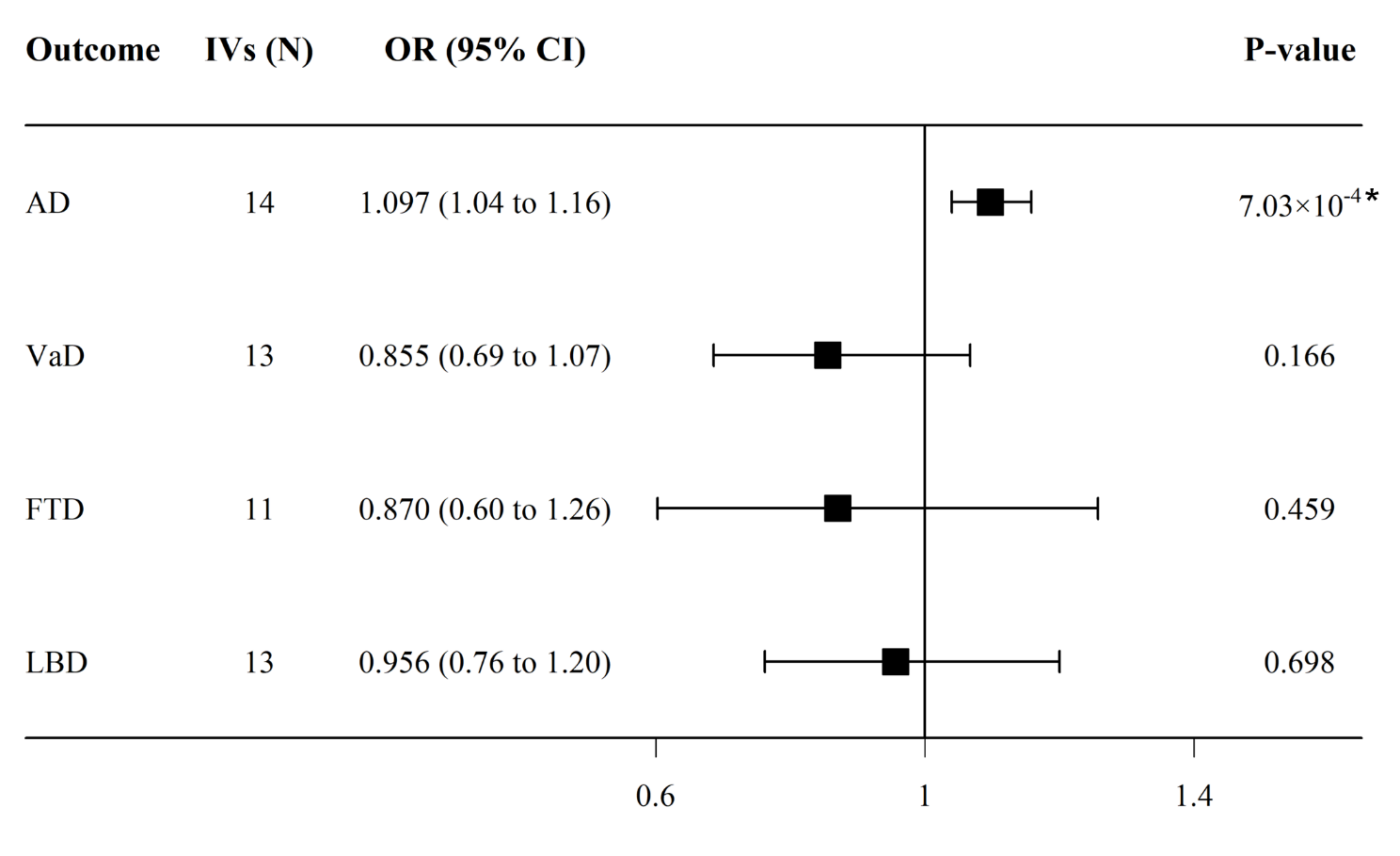
Causal effects of migraine on four common types of dementia. *Abbreviations* AD, Alzheimer’s disease; VaD, vascular dementia; FTD, frontotemporal dementia; LBD, Lewy body dementia; IVs, instrumental variables; OR, odds ratio; CI, confidence interval. *denotes *p* < 0.0125 at Bonferroni correction

As shown in Fig. 2, genetically predicted migraine was significantly associated with an increased risk of AD (OR = 1.097, 95% confidence interval (CI) = [1.040, 1.158], *p* = 7.03 × 10^− 4^). There was no evidence of a causal effect of migraine on VaD (OR = 0.855, 95% CI = [0.686, 1.067], *p* = 0.166), FTD (OR = 0.870, 95% CI = [0.602, 1.257], *p* = 0.459), or LBD (OR = 0.956, 95% CI = [0.762, 1.200], *p* = 0.698).

### The putative causal effects of migraine on longitudinal brain measures

**Fig. 3:**
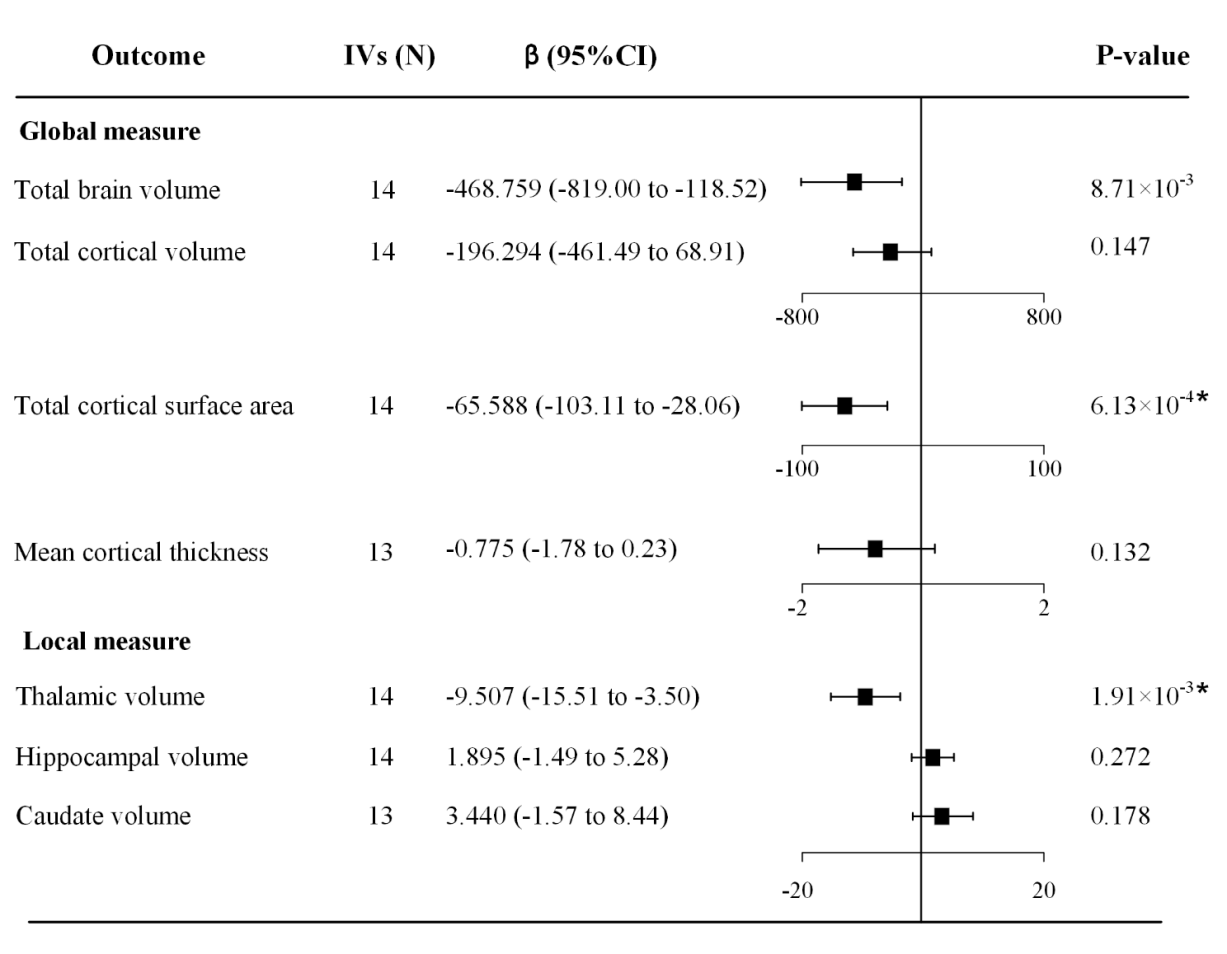
Causal effects of migraine on seven longitudinal brain measures. *Abbreviations* IVs, instrumental variables; CI, confidence interval. *denotes *p* < 0.0071 at Bonferroni correction

As shown in Fig. 3, migraine showed significant causal effects on annual changes of the total cortical surface area (*β* = -65.588, 95% CI = [-103.112, -28.064], *p* = 6.13 × 10^− 4^) and thalamic volume (*β* = -9.507, 95% CI = [-15.512, -3.502], *p* = 1.91 × 10^− 3^). Specifically, genetically predicted migraine was associated with an accelerated atrophy of the total cortical surface area, resulting in a decrease of 65.588 cm^2^ per year, as well as a reduction in thalamic volume by 9.507 cm^3^ per year.

### The putative causal effects of migraine subtypes on AD and longitudinal brain measures

**Fig. 4:**
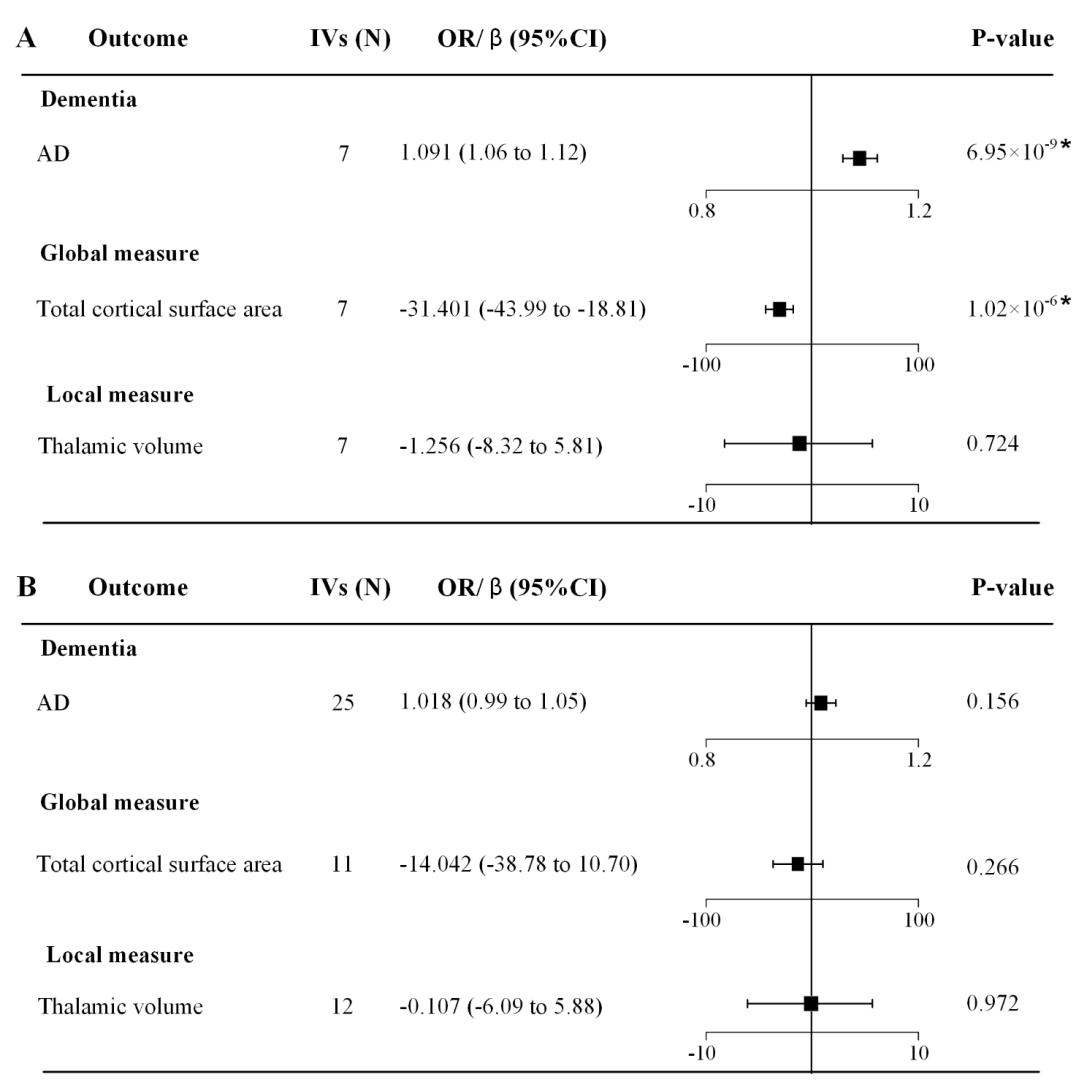
Replications of significant MR estimates in two migraine subtypes. (**A**) MR analysis for the MO subtype (**B**) MR analysis for the MA subtype. *Abbreviations* AD, Alzheimer’s disease; IVs, instrumental variables; OR, odds ratio; CI, confidence interval. *denotes *p* < 0.0083 at Bonferroni correction

Consistent with migraine, the MO subtype was significantly associated with an increased risk of AD (OR = 1.091, 95% CI = [1.059, 1.123], *p* = 6.95 × 10^− 9^; Fig. 4A). For longitudinal brain measures, the MO subtype accelerated annual atrophy of the total cortical surface area, with a decrease of 31.401 cm [2] per year (*β* = -31.401, 95% CI = [-43.990, -18.811], *p* = 1.02 × 10^− 6^; Fig. 4A). However, there was no evidence of causal relationships (Fig. 4B) between the MA subtype and AD (OR = 1.018, 95% CI = [0.992, 1.045], *p* = 0.156) and longitudinal brain measures (*p* > 0.05). Comprehensive details regarding the SNPs utilized for MR estimations have been incorporated into Tables S1-3.

### Results of sensitivity analysis

As shown in Tables S4-8, no evidence of heterogeneity or horizontal pleiotropy (*p* > 0.05) was found. Moreover, the causal estimates were not driven by any single genetic IV (Figures S1-6). In Phenoscanner, two genetic IVs (rs10456100, rs9349379) of migraine were associated with coronary artery disease, and one (rs11187838) was associated with hypertension at a genome-wide significant threshold (*p* < 5 × 10^− 8^). One genetic IV (rs9349379) of MO was associated with coronary artery disease at a genome-wide significant threshold (*p* < 5 × 10^− 8^). Nevertheless, the causal estimates were not affected by the removal of these genetic IVs (Figures S7-8), excluding the potential horizontal pleiotropy. We used three supplementary MR methods to examine the robustness of the IVW method and demonstrated that the directions estimated by supplementary MR methods were consistent with the findings in the IVW method (Figures S9-14). The reversed MR analysis did not provide evidence supporting the causal effects of AD on migraine (Figure S15). Though the remaining four local brain measures provided by Brouwer et al. were not the primary focus of our current investigation [28], we evaluated and displayed their respective causal associations with migraine in Figure S16.

### Mediation analysis

**Fig. 5:**
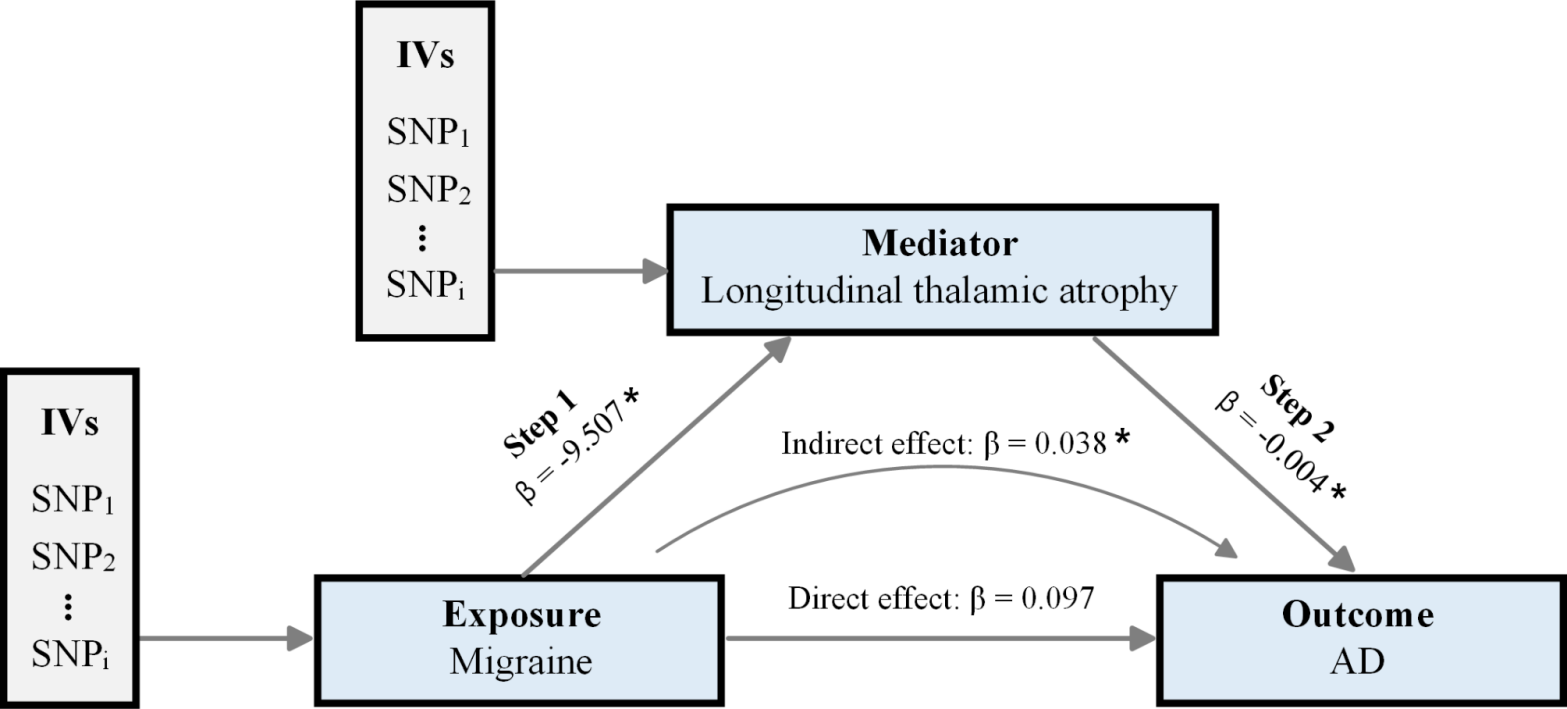
Mediation effect of migraine on AD via longitudinal thalamic atrophy. *Abbreviations* AD, Alzheimer’s disease; IVs, instrumental variables; SNP, Single Nucleotide Polymorphism. *denotes *p* < 0.05

Consistent with the primary findings, migraine was significantly associated with an increased risk of AD (OR = 1.145, 95% CI = [1.052, 1.246], *p* = 1.63 × 10^− 3^) when using the GWAS of AD in the FinnGen database. In addition, annual atrophy of the thalamic volume significantly increased the risk of AD (OR = 0.996, 95% CI = [0.993, 0.999], *p* = 6.28 × 10^− 3^). No significant association was found between annual atrophy of the total cortical surface area and risk of AD (OR = 1.000, 95% CI = [0.999, 1.001], *p* = 0.707). As shown in Fig. 5, the mediation analysis demonstrated that annual atrophy of the thalamic volume showed a significant mediation effect between migraine and AD (*β* = 0.038, CI = [0.002, 0.074], *p* = 0.040), with a mediated proportion of 28.2%.

## Discussion

In this study, we used genetic variants as IVs of MR to investigate the causal effects of migraine on four common types of dementia and seven longitudinal brain measures. Migraine significantly increased the risk of AD and accelerated annual atrophy of the total cortical surface area and thalamic volume. We also found that annual atrophy of the thalamic volume partially mediated the effect of migraine on AD. In migraine subtypes analysis, only MO had causal relationships with AD and annual atrophy of the total cortical surface area, while MA did not show any significant association.

Migraine has been recognized as a midlife risk factor for the development of dementia [5]. AD is the most prevalent type of dementia and the most frequently reported outcome of migraine among common types of dementia [3, 4, 20, 21]. Nonetheless, migraine and AD share some risk factors such as smoking, drinking, major depressive disorder, and hypertension, which may introduce spurious associations in observational studies. (40–41) Using genetic variants and MR analysis, we identified a significant causal effect of migraine on AD, but not on the other three types of dementia. The genetically determined migraine was associated with approximately 10% higher odds of AD risk. The association between migraine and VaD, the second most common type of dementia, is controversial in observational studies [4, 20–22]. Previous studies reported a higher risk of VaD in individuals with migraine compared to those without migraine [4, 22]. Two recent longitudinal studies suggested that individuals with either self-reported or diagnosed migraine have an increased risk of AD in the future, but not VaD. (20–21) Nevertheless, our findings indicated that there was no genetic causal association between migraine and VaD.

Two recent MR studies have explored the causal effects of migraine on brain measures, (42–43) but the GWAS data in these studies only assessed the genetic contributions to cross-sectional variations in brain structures. Using the GWAS of longitudinal brain measures from MRI data [28], we found that migraine caused faster atrophy of the total cortical surface area and thalamic volume. These associations were not found in previous MR studies using the GWAS of cross-sectional brain measures, (42–43) suggesting that migraine may not cause a fixed level of brain atrophy that is independent of the aging process, but rather affect the rate and pattern of brain atrophy that occurs as a person ages. We found causal effects of migraine on annual atrophy of the cortical surface area, but not on cortical volume and thickness. This is consistent with neuroimaging studies that reported more widespread atrophy in the cortical surface area than the other two measures in migraine patients. (44–45)

Thalamic atrophy is heterogeneous and can be attributed to various diseases or risk factors [46]. Several studies have shown that migraine patients have faster thalamic atrophy than age-matched healthy participants [9, 11]. The thalamus is involved in pain processing and consistently activated in response to painful stimuli across multiple human imaging studies [47]. Migraine is characterized by long-lasting episodes of headache and showed abnormal neural activity in the thalamus [48], implying that headache episodes may damage the thalamus. We conducted a two-step MR mediation analysis and found that a faster annual thalamic atrophy partly mediated the causal effect of migraine on AD. In addition to pain processing, the thalamus is also a key component for cognitive functions that decline with aging, such as memory, attention, and executive functions [49]. It connects with cortical and subcortical regions and is involved in the pathogenesis of neurodegenerative disorders. Several studies have reported reduced thalamic volume and impaired cognitive function in AD [12, 50, 51]. Moreover, some recent findings demonstrated that thalamic atrophy precedes the onset of AD and occurs in mild cognitive impairment patients, highlighting the role of thalamic atrophy in the development of AD. (52–53) Overall, our results suggest that migraine leads to an increased risk of AD by influencing the atrophy trajectory of the thalamus with age. This atrophy is persistent instead of temporary and thus leaves precious opportunity for intervention for AD risk in migraine patients.

We also found causal associations between MO and AD and the accelerated atrophy of the cortical surface area. However, we did not find a causal effect of MA on AD, contrary to previous observational studies. (5–6) This finding is unlikely to be due to statistical power, as the GWAS of two migraine subtypes had comparable sample sizes. The comorbidities may explain this inconsistency. Compared to MO, patients with MA may have a higher prevalence of comorbid conditions known to be risk factors for AD, such as stroke and coronary artery disease [54]. The presence of these comorbidities in MA may inflate the associations between MA and AD in observational studies.

The study has several limitations. First, although using the largest available GWAS, the sample sizes for FTD and LBD were limited, which may affect the statistical power of the MR estimates. Second, all GWAS in this study was based on participants of European ancestry, which reduced the bias from population stratification but also limited the generalizability to other populations. Third, the GWAS in this study were not sex-specific, although sex differences exist in migraine, brain structures, and AD. This may prevent the detection of sex-specific causal relationships.

In conclusion, this study provided genetic evidence supporting a causal link between migraine and AD, with a faster annual atrophy of the thalamus serving as a mediator of this association. These findings will encourage further investigation of the causal effect of migraine on AD and contribute to a better intervention targeting the potential AD risk in patients with migraine at the neural level.

### Electronic supplementary material

Below is the link to the electronic supplementary material.

Supplementary Material 1

## Data Availability

No datasets were generated or analysed during the current study.
