# Supplementary material for: Genetic evidence for the causal relationships between migraine, dementia, and longitudinal brain atrophy
Source: J Headache Pain. 2024 Jun 5;25(1):93. doi: 10.1186/s10194-024-01801-7 (PMC11151614; doi:10.1186/s10194-024-01801-7)
Supplement: Supplementary file 1 — Supplementary Material 1 [file 10194_2024_1801_MOESM1_ESM.docx]

**Table S1. The SNPs used for MR analysis between migraine and dementia**

| **No.** | **SNP** | **Effect allele** | **Reference allele** |
| --- | --- | --- | --- |
| **Exposure (Migraine); Outcome (AD)** | | | |
| 1 | Rs10166942 | T | C |
| 2 | Rs10218452 | A | G |
| 3 | Rs10234636 | C | T |
| 4 | Rs10456100 | C | T |
| 5 | Rs10849061 | C | T |
| 6 | Rs11153082 | A | G |
| 7 | Rs11172113 | T | C |
| 8 | Rs11187838 | G | A |
| 9 | Rs2078371 | T | C |
| 10 | Rs2274319 | C | T |
| 11 | Rs34979631 | C | T |
| 12 | Rs42854 | C | G |
| 13 | Rs4910165 | G | C |
| 14 | Rs9349379 | A | G |
| **Exposure (Migraine); Outcome (VaD)** | | | |
| 1 | Rs10166942 | T | C |
| 2 | Rs10218452 | A | G |
| 3 | Rs10234636 | C | T |
| 4 | Rs10456100 | C | T |
| 5 | Rs10849061 | C | T |
| 6 | Rs11153082 | A | G |
| 7 | Rs11172113 | T | C |
| 8 | Rs11187838 | G | A |
| 9 | Rs2078371 | T | C |
| 10 | Rs2274319 | C | T |
| 11 | Rs34979631 | C | T |
| 12 | Rs4910165 | G | C |
| 13 | Rs9349379 | A | G |
| **Exposure (Migraine); Outcome (FTD)** | | | |
| 1 | Rs10166942 | T | C |
| 2 | Rs10218452 | A | G |
| 3 | Rs10234636 | C | T |
| 4 | Rs10849061 | C | T |
| 5 | Rs11153082 | A | G |
| 6 | Rs11172113 | T | C |
| 7 | Rs11187838 | G | A |
| 8 | Rs2078371 | T | C |
| 9 | Rs2274319 | C | T |
| 10 | Rs34979631 | C | T |
| 11 | Rs9349379 | A | G |
| **Exposure (Migraine); Outcome (LBD)** | | | |
| 1 | Rs10166942 | T | C |
| 2 | Rs10218452 | A | G |
| 3 | Rs10234636 | C | T |
| 4 | Rs10456100 | C | T |
| 5 | Rs10849061 | C | T |
| 6 | Rs11153082 | A | G |
| 7 | Rs11172113 | T | C |
| 8 | Rs11187838 | G | A |
| 9 | Rs2078371 | T | C |
| 10 | Rs2274319 | C | T |
| 11 | Rs34979631 | C | T |
| 12 | Rs4910165 | G | C |
| 13 | Rs9349379 | A | G |

**Abbreviations:** AD, Alzheimer's disease; VaD, vascular dementia; FTD, frontotemporal dementia; LBD, Lewy body dementia; SNP, Single Nucleotide Polymorphism.

**Table S2. The SNPs used for MR analysis between migraine and longitudinal brain measures**

| **No.** | **SNP** | **Effect allele** | **Reference allele** |
| --- | --- | --- | --- |
| **Exposure (Migraine); Outcome (Total brain volume)** | | | |
| 1 | Rs10166942 | T | C |
| 2 | Rs10218452 | A | G |
| 3 | Rs10234636 | C | T |
| 4 | Rs10456100 | C | T |
| 5 | Rs10849061 | C | T |
| 6 | Rs11153082 | A | G |
| 7 | Rs11172113 | T | C |
| 8 | Rs11187838 | G | A |
| 9 | Rs145639541 | T | C |
| 10 | Rs2078371 | T | C |
| 11 | Rs2274319 | C | T |
| 12 | Rs34979631 | C | T |
| 13 | Rs4910165 | G | C |
| 14 | Rs9349379 | A | G |
| **Exposure (Migraine); Outcome (Total cortical volume)** | | | |
| 1 | Rs10166942 | T | C |
| 2 | Rs10218452 | A | G |
| 3 | Rs10234636 | C | T |
| 4 | Rs10456100 | C | T |
| 5 | Rs10849061 | C | T |
| 6 | Rs11153082 | A | G |
| 7 | Rs11172113 | T | C |
| 8 | Rs11187838 | G | A |
| 9 | Rs145639541 | T | C |
| 10 | Rs2078371 | T | C |
| 11 | Rs2274319 | C | T |
| 12 | Rs34979631 | C | T |
| 13 | Rs4910165 | G | C |
| 14 | Rs9349379 | A | G |
| **Exposure (Migraine); Outcome (Total cortical surface area)** | | | |
| 1 | Rs10166942 | T | C |
| 2 | Rs10218452 | A | G |
| 3 | Rs10234636 | C | T |
| 4 | Rs10456100 | C | T |
| 5 | Rs10849061 | C | T |
| 6 | Rs11153082 | A | G |
| 7 | Rs11172113 | T | C |
| 8 | Rs11187838 | G | A |
| 9 | Rs145639541 | T | C |
| 10 | Rs2078371 | T | C |
| 11 | Rs2274319 | C | T |
| 12 | Rs34979631 | C | T |
| 13 | Rs4910165 | G | C |
| 14 | Rs9349379 | A | G |
| **Exposure (Migraine); Outcome (Mean cortical thickness)** | | | |
| 1 | Rs10166942 | T | C |
| 2 | Rs10218452 | A | G |
| 3 | Rs10234636 | C | T |
| 4 | Rs10456100 | C | T |
| 5 | Rs10849061 | C | T |
| 6 | Rs11153082 | A | G |
| 7 | Rs11172113 | T | C |
| 8 | Rs11187838 | G | A |
| 9 | Rs2078371 | T | C |
| 10 | Rs2274319 | C | T |
| 11 | Rs34979631 | C | T |
| 12 | Rs4910165 | G | C |
| 13 | Rs9349379 | A | G |
| **Exposure (Migraine); Outcome (Caudate volume)** | | | |
| 1 | Rs10166942 | T | C |
| 2 | Rs10218452 | A | G |
| 3 | Rs10234636 | C | T |
| 4 | Rs10456100 | C | T |
| 5 | Rs10849061 | C | T |
| 6 | Rs11153082 | A | G |
| 7 | Rs11172113 | T | C |
| 8 | Rs145639541 | T | C |
| 9 | Rs2078371 | T | C |
| 10 | Rs2274319 | C | T |
| 11 | Rs34979631 | C | T |
| 13 | Rs4910165 | G | C |
| 14 | Rs9349379 | A | G |
| **Exposure (Migraine); Outcome (Hippocampal volume)** | | | |
| 1 | Rs10166942 | T | C |
| 2 | Rs10218452 | A | G |
| 3 | Rs10234636 | C | T |
| 4 | Rs10456100 | C | T |
| 5 | Rs10849061 | C | T |
| 6 | Rs11153082 | A | G |
| 7 | Rs11172113 | T | C |
| 8 | Rs11187838 | G | A |
| 9 | Rs145639541 | T | C |
| 10 | Rs2078371 | T | C |
| 11 | Rs2274319 | C | T |
| 12 | Rs34979631 | C | T |
| 13 | Rs4910165 | G | C |
| 14 | Rs9349379 | A | G |
| **Exposure (Migraine); Outcome (Thalamic volume)** | | | |
| 1 | Rs10166942 | T | C |
| 2 | Rs10218452 | A | G |
| 3 | Rs10234636 | C | T |
| 4 | Rs10456100 | C | T |
| 5 | Rs10849061 | C | T |
| 6 | Rs11153082 | A | G |
| 7 | Rs11172113 | T | C |
| 8 | Rs11187838 | G | A |
| 9 | Rs145639541 | T | C |
| 10 | Rs2078371 | T | C |
| 11 | Rs2274319 | C | T |
| 12 | Rs34979631 | C | T |
| 13 | Rs4910165 | G | C |
| 14 | Rs9349379 | A | G |

**Abbreviations:** SNP, Single Nucleotide Polymorphism.

**Table S3. The SNPs used for MR analysis between migraine subtypes, dementia and longitudinal brain measures**

| **No.** | **SNP** | **Effect allele** | **Reference allele** |
| --- | --- | --- | --- |
| **Exposure (MO); Outcome (AD)** | | | |
| 1 | Rs11172113 | C | T |
| 2 | Rs2078371 | C | T |
| 3 | Rs2160875 | T | C |
| 4 | Rs6478241 | G | A |
| 5 | Rs6724624 | G | C |
| 6 | Rs7775721 | T | C |
| 7 | Rs9349379 | G | A |
| **Exposure (MO); Outcome (Total cortical surface area)** | | | |
| 1 | Rs11172113 | C | T |
| 2 | Rs2078371 | C | T |
| 3 | Rs2160875 | T | C |
| 4 | Rs6478241 | G | A |
| 5 | Rs6724624 | G | C |
| 6 | Rs7775721 | T | C |
| 7 | Rs9349379 | G | A |
| **Exposure (MO); Outcome (Thalamic volume)** | | | |
| 1 | Rs11172113 | C | T |
| 2 | Rs2078371 | C | T |
| 3 | Rs2160875 | T | C |
| 4 | Rs6478241 | G | A |
| 5 | Rs6724624 | G | C |
| 6 | Rs7775721 | T | C |
| 7 | Rs9349379 | G | A |
| **Exposure (MA); Outcome (AD)** | | | |
| 1 | rs10812660 | A | T |
| 2 | rs111622936 | T | C |
| 3 | rs11172113 | C | T |
| 4 | rs115695766 | C | T |
| 5 | rs1157316 | A | G |
| 6 | rs116487794 | T | G |
| 7 | rs11702060 | C | A |
| 8 | rs117067760 | A | G |
| 9 | rs117727533 | A | G |
| 10 | rs12967385 | C | T |
| 11 | rs138553949 | A | G |
| 12 | rs140390695 | A | G |
| 13 | rs143910632 | C | T |
| 14 | rs146241454 | T | C |
| 15 | rs17887050 | A | C |
| 16 | rs1832937 | A | G |
| 17 | rs2225164 | A | G |
| 18 | rs2506145 | C | T |
| 19 | rs35695296 | A | T |
| 20 | rs4708524 | T | G |
| 21 | rs4910165 | G | C |
| 22 | rs61945078 | C | T |
| 23 | rs6535076 | C | T |
| 24 | rs72738438 | C | G |
| 25 | rs75879915 | T | C |
| **Exposure (MA); Outcome (Total cortical surface area)** | | | |
| 1 | Rs11172113 | C | T |
| 2 | rs1157316 | A | G |
| 3 | rs11702060 | C | A |
| 4 | rs12967385 | C | T |
| 5 | rs12988953 | G | C |
| 6 | rs1832937 | A | G |
| 7 | rs2506145 | C | T |
| 8 | rs35695296 | A | T |
| 9 | rs4910165 | G | C |
| 10 | rs6535076 | C | T |
| 11 | rs7713256 | T | C |
| **Exposure (MA); Outcome (Thalamic volume)** | | | |
| 1 | Rs11172113 | C | T |
| 2 | rs1157316 | A | G |
| 3 | rs11702060 | C | A |
| 4 | rs12967385 | C | T |
| 5 | rs12988953 | G | C |
| 6 | rs1832937 | A | G |
| 7 | rs2506145 | C | T |
| 8 | rs35695296 | A | T |
| 9 | rs4708524 | T | G |
| 10 | rs4910165 | G | C |
| 11 | rs6535076 | C | T |
| 12 | rs7713256 | T | C |

**Abbreviations:** AD, Alzheimer's disease; SNP, Single Nucleotide Polymorphism; MA, migraine with aura; MO, migraine without aura.

**Table S4. The sensitivity analysis of MR analysis between migraine and dementia**

| **Exposure** | **Outcome** | **MR-egger regression** | **MR-PRESSO Global test** | **Cochran’s Q-test** |
| --- | --- | --- | --- | --- |
| Migraine | AD | Intercept = 0.01  *p* = 0.921 | *RSS* = 12.87  *p* = 0.614 | *Q*-value = 11.17  *p* = 0.597 |
|  | VaD | Intercept = 0.02  *p* = 0.694 | *RSS* = 13.12  *p* = 0.532 | *Q*-value = 10.67  *p* = 0.557 |
|  | FTD | Intercept = 0.09  *p* = 0.212 | *RSS* = 16.82  *p* = 0.185 | *Q*-value = 11.17  *p* = 0.202 |
|  | LBD | Intercept = 0.08  *p* = 0.166 | *RSS* = 10.25  *p* = 0.728 | *Q*-value = 8.56  *p* = 0.740 |

**Abbreviations:** AD, Alzheimer's disease; VaD, vascular dementia; FTD, frontotemporal dementia; LBD, Lewy body dementia; RSS, residual sum of squares.

**Table S5. The sensitivity analysis of MR analysis between migraine and longitudinal brain measures**

| **Exposure** | **Outcome** | **MR-egger regression** | **MR-PRESSO**  **Global test** | **Cochran’s**  **Q-test** |
| --- | --- | --- | --- | --- |
| Migraine | Total brain volume | Intercept = -0.55  *p* = 0.994 | *RSS* = 12.35  *p* = 0.662 | *Q*-value = 10.52  *p* = 0.651 |
|  | Total cortical volume | Intercept = 43.15  *p* = 0.516 | *RSS* = 17.82  *p* = 0.287 | *Q*-value = 15.32  *p* = 0.288 |
|  | Total cortical surface area | Intercept = -9.21  *p* = 0.311 | *RSS* = 9.90  *p* = 0.834 | *Q*-value = 8.79  *p* = 0.788 |
|  | Mean cortical thickness | Intercept = 0.04  *p* = 0.866 | *RSS* = 9.51  *p* = 0.793 | *Q*-value = 8.19  *p* = 0.770 |
|  | Thalamic volume | Intercept = 0.71  *p* = 0.672 | *RSS* = 7.24  *p* = 0.945 | *Q*-value = 6.35  *p* = 0.932 |
|  | Hippocampal volume | Intercept = -0.60  *p* = 0.612 | *RSS* = 4.85  *p* = 0.987 | *Q*-value = 4.11  *p* = 0.990 |
|  | Caudate volume | Intercept = 0.53  *p* = 0.644 | *RSS* = 11.38  *p* = 0.616 | *Q*-value = 9.80  *p* = 0.633 |

**Abbreviations:** RSS, residual sum of squares.

**Table S6. The sensitivity analysis of MR analysis between migraine subtypes and AD**

| **Exposure** | **Outcome** | **MR-egger regression** | **MR-PRESSO Global test** | **Cochran’s Q-test** |
| --- | --- | --- | --- | --- |
| MA | AD | Intercept = 0.01  *p* = 0.225 | *RSS* =  20.90  *p* = 0.741 | *Q*-value = 19.25  *p* = 0.739 |
| MO |  | Intercept = -0.01  *p* = 0.987 | *RSS* =  3.03  *p* = 0.908 | *Q*-value = 2.25  *p* = 0.895 |

**Abbreviations:** AD, Alzheimer's disease; MA, migraine with aura; MO, migraine without aura; RSS, residual sum of squares.

**Table S7. The sensitivity analysis of MR analysis between migraine subtypes and longitudinal brain measures**

| **Exposure** | **Outcome** | **MR-egger regression** | **MR-PRESSO**  **Global test** | **Cochran’s**  **Q-test** |
| --- | --- | --- | --- | --- |
| MA | Total cortical surface area | Intercept = -7.27  *p* = 0.575 | *RSS* = 5.38  *p* = 0.929 | *Q*-value = 4.45  *p* = 0.925 |
|  | Thalamic volume | Intercept = 0.26  *p* = 0.912 | *RSS* = 10.59  *p* = 0.627 | *Q*-value = 8.86  *p* = 0.635 |
| MO | Total cortical surface area | Intercept = 4.07  *p* = 0.855 | *RSS* = 0.88  *p* = 0.998 | *Q*-value = 0.66  *p* = 0.995 |
|  | Thalamic volume | Intercept = 3.45  *p* = 0.430 | *RSS* = 7.82  *p* = 0.468 | *Q*-value = 5.83  *p* = 0.442 |

**Abbreviations:** MA, migraine with aura; MO, migraine without aura; RSS, residual sum of squares.

| **Exposure** | **Outcome** | **MR-egger regression** | **MR-PRESSO Global test** | **Cochran’s Q-test** |
| --- | --- | --- | --- | --- |
| Migraine | AD | Intercept = 0.02  *p* = 0.441 | *RSS* = 8.55  *p* = 0.886 | *Q*-value = 7.32  *p* = 0.885 |

**Table S8. The sensitivity analysis of MR analysis between migraine and AD using the GWAS of AD in the FinnGen database**

**Abbreviations:** AD, Alzheimer's disease; RSS, residual sum of squares.

**Figure S1. LOO sensitivity analysis of MR analysis between migraine and AD**


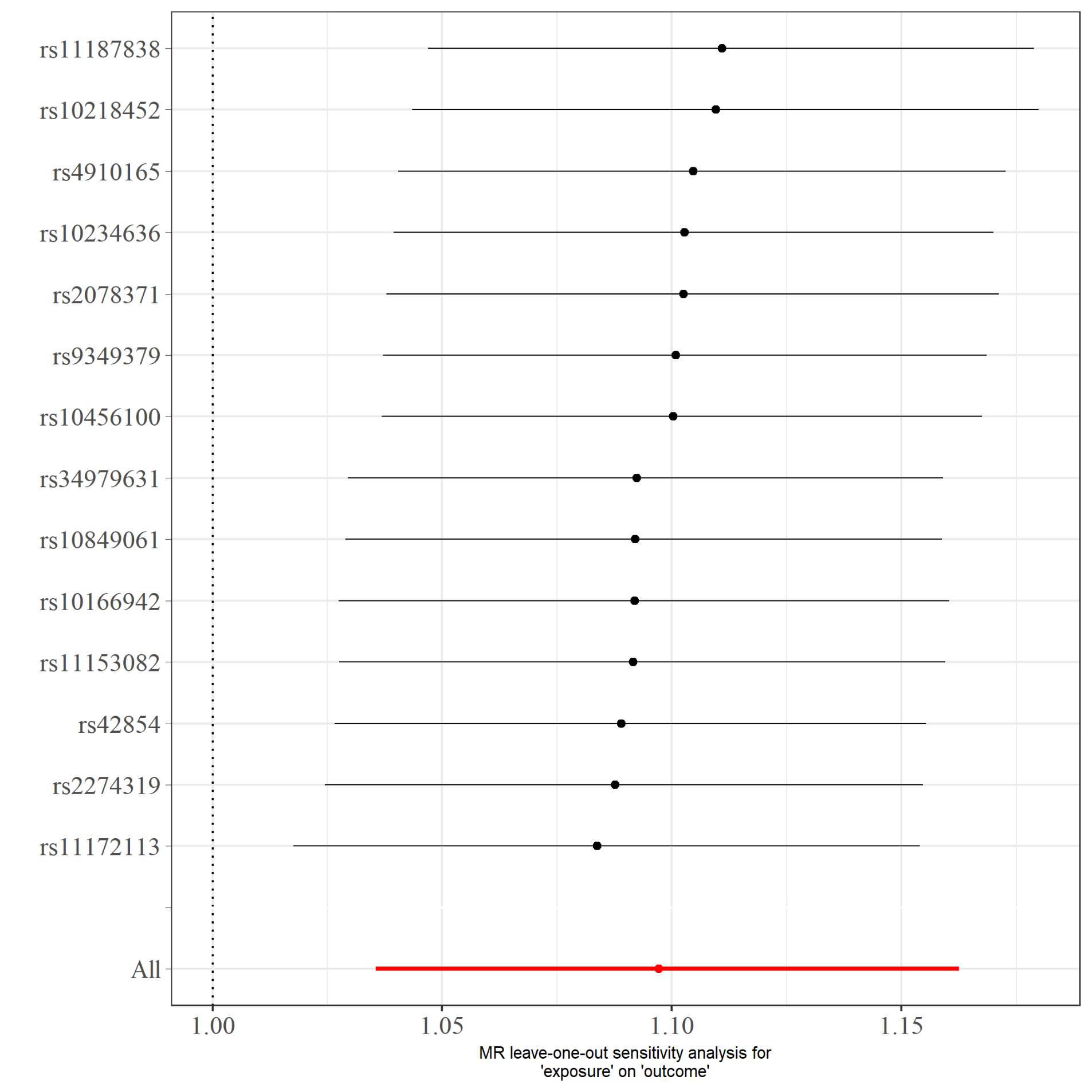


**Figure S2. LOO sensitivity analysis of MR analysis between migraine and total cortical surface area
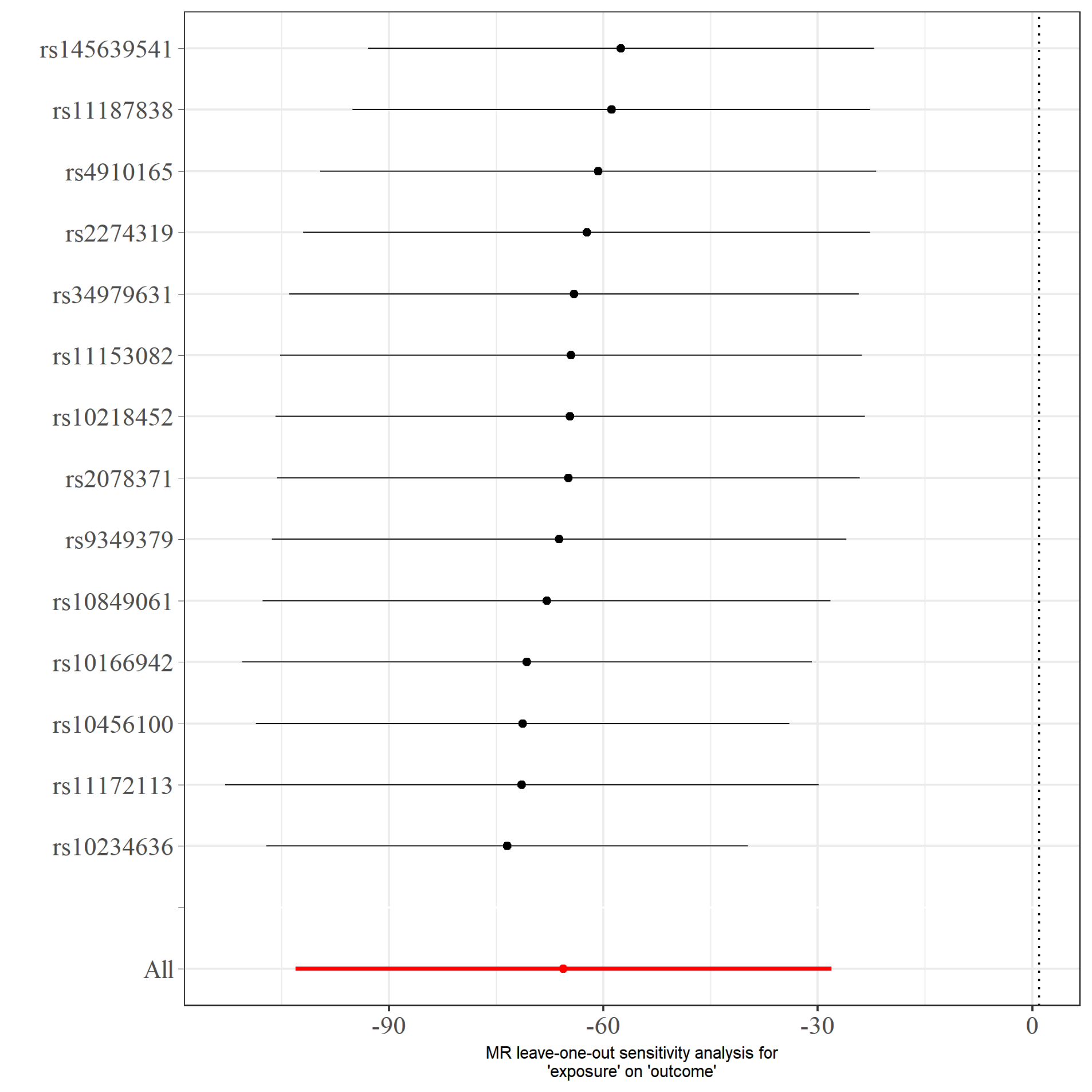
**

**Figure S3. LOO sensitivity analysis of MR analysis between migraine and thalamic volume**


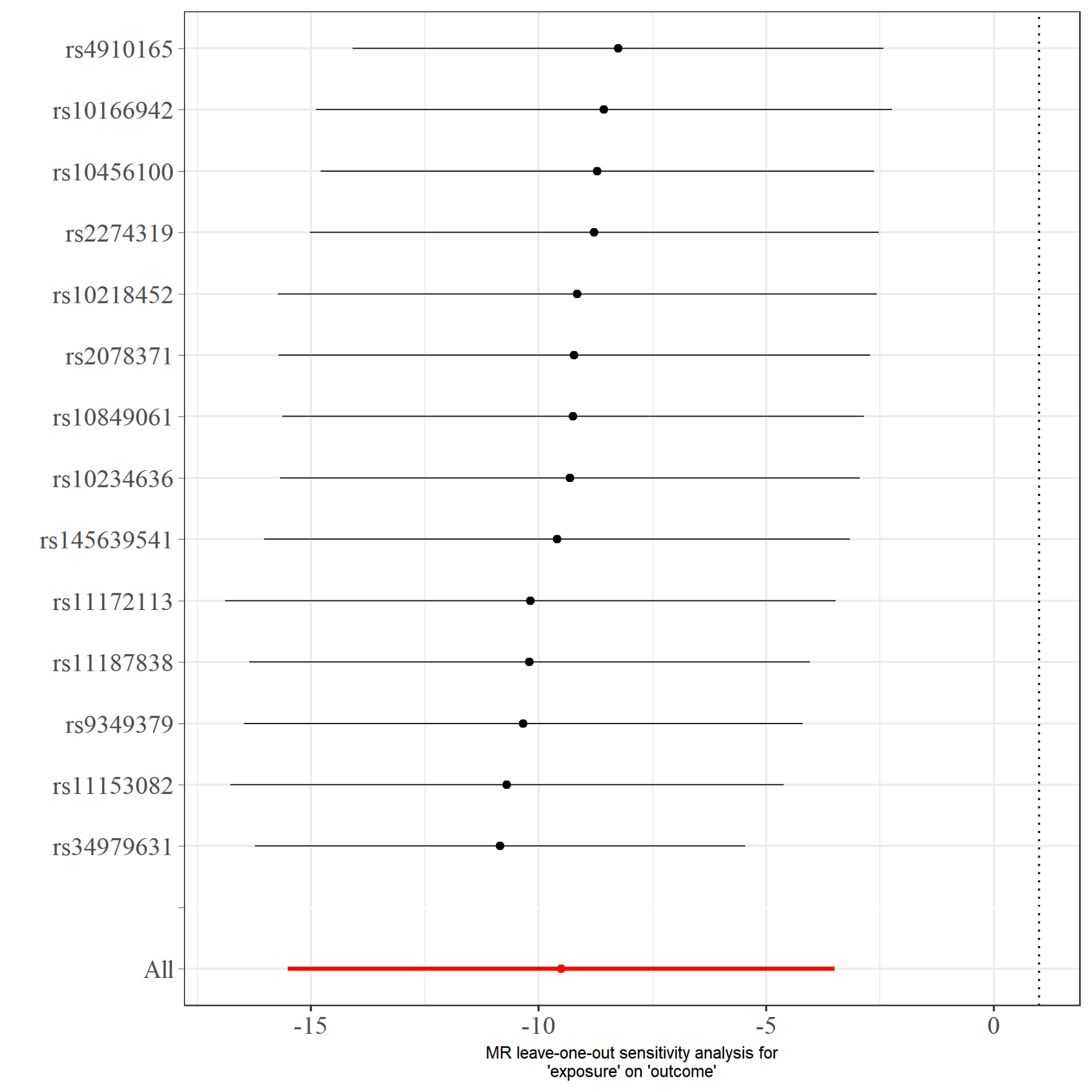


**Figure S4. LOO sensitivity analysis of MR analysis between MO and AD**


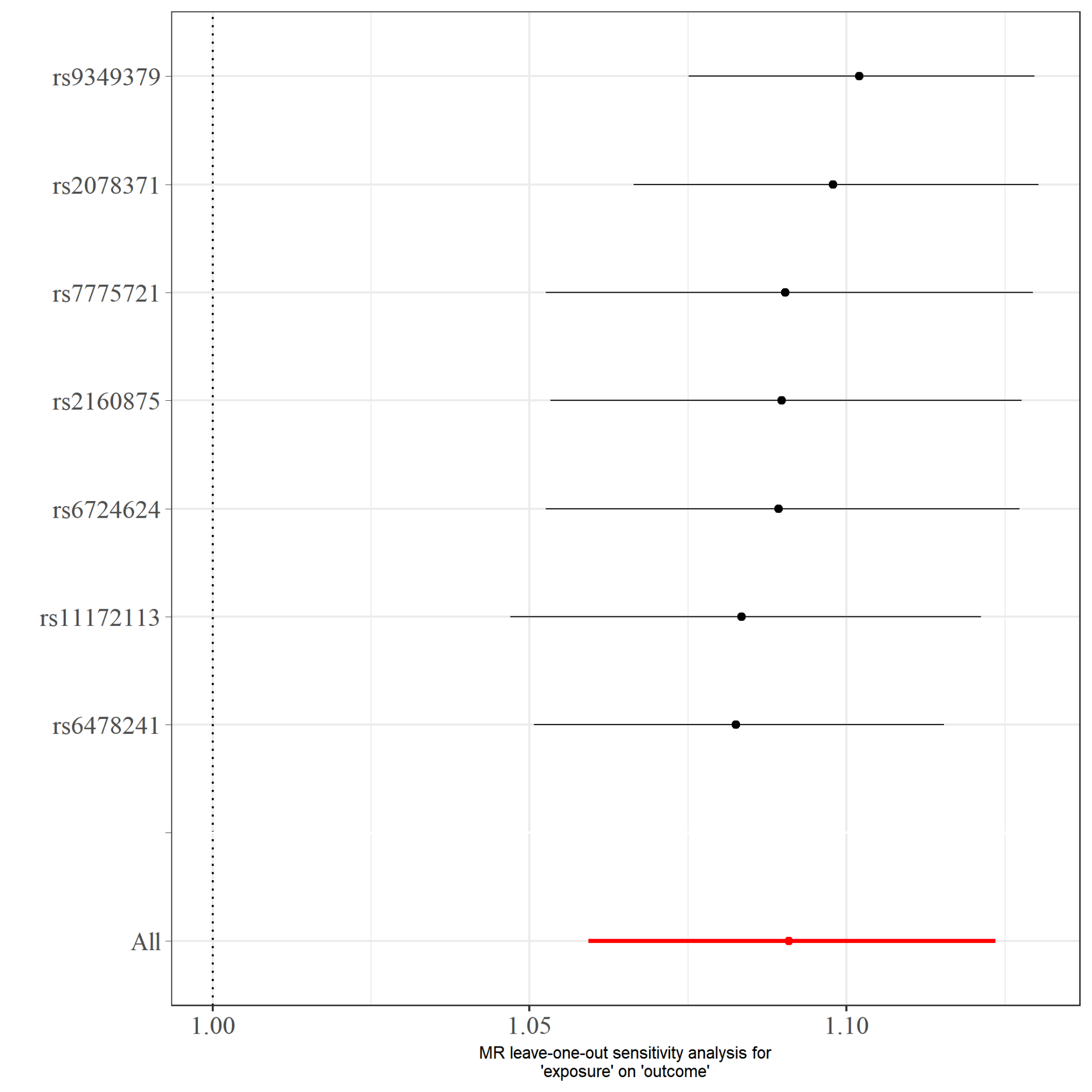


**Figure S5. LOO sensitivity analysis of MR analysis between MO and total cortical surface area**


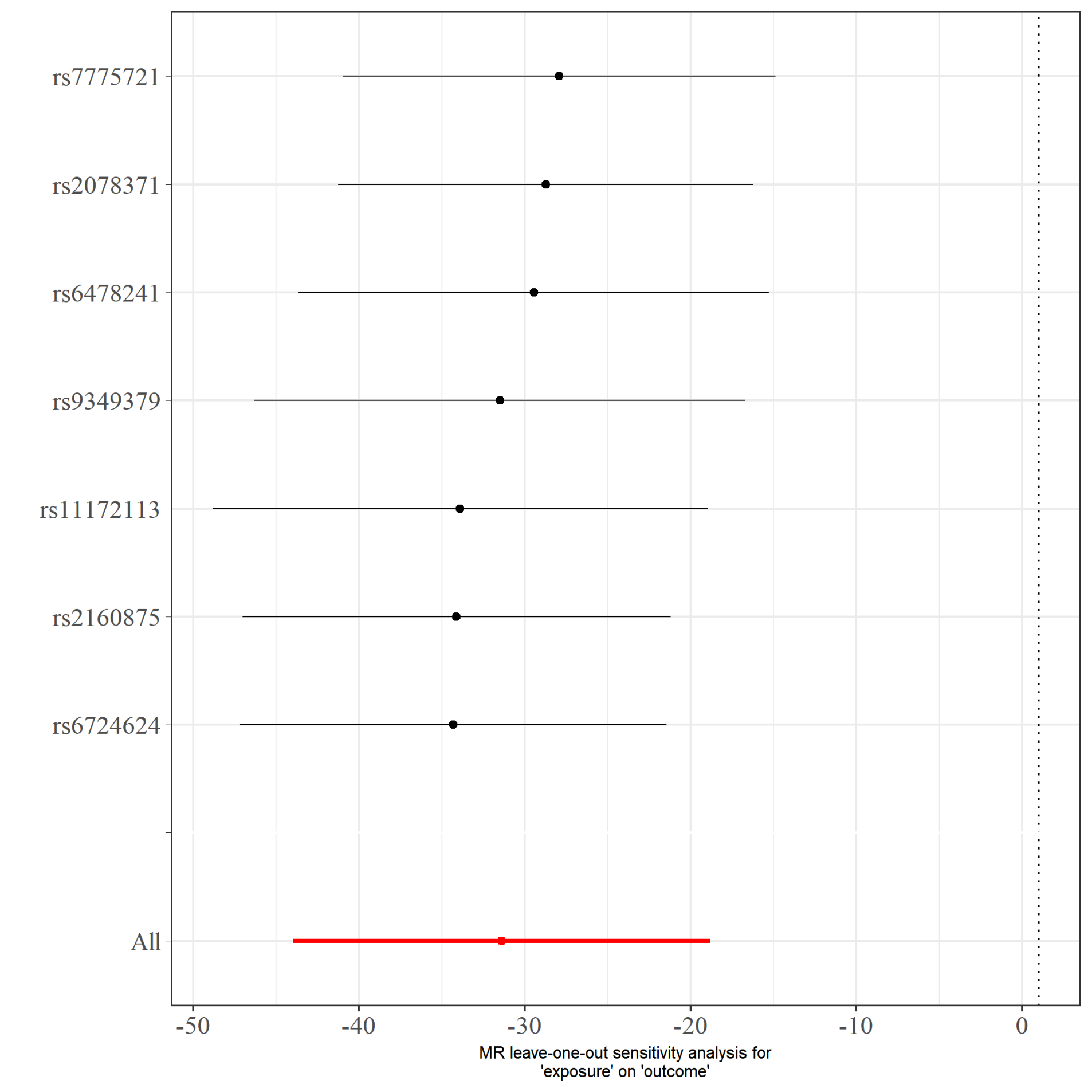


**Figure S6. LOO sensitivity analysis of MR analysis between migraine and AD using the GWAS of AD in the FinnGen database**

**
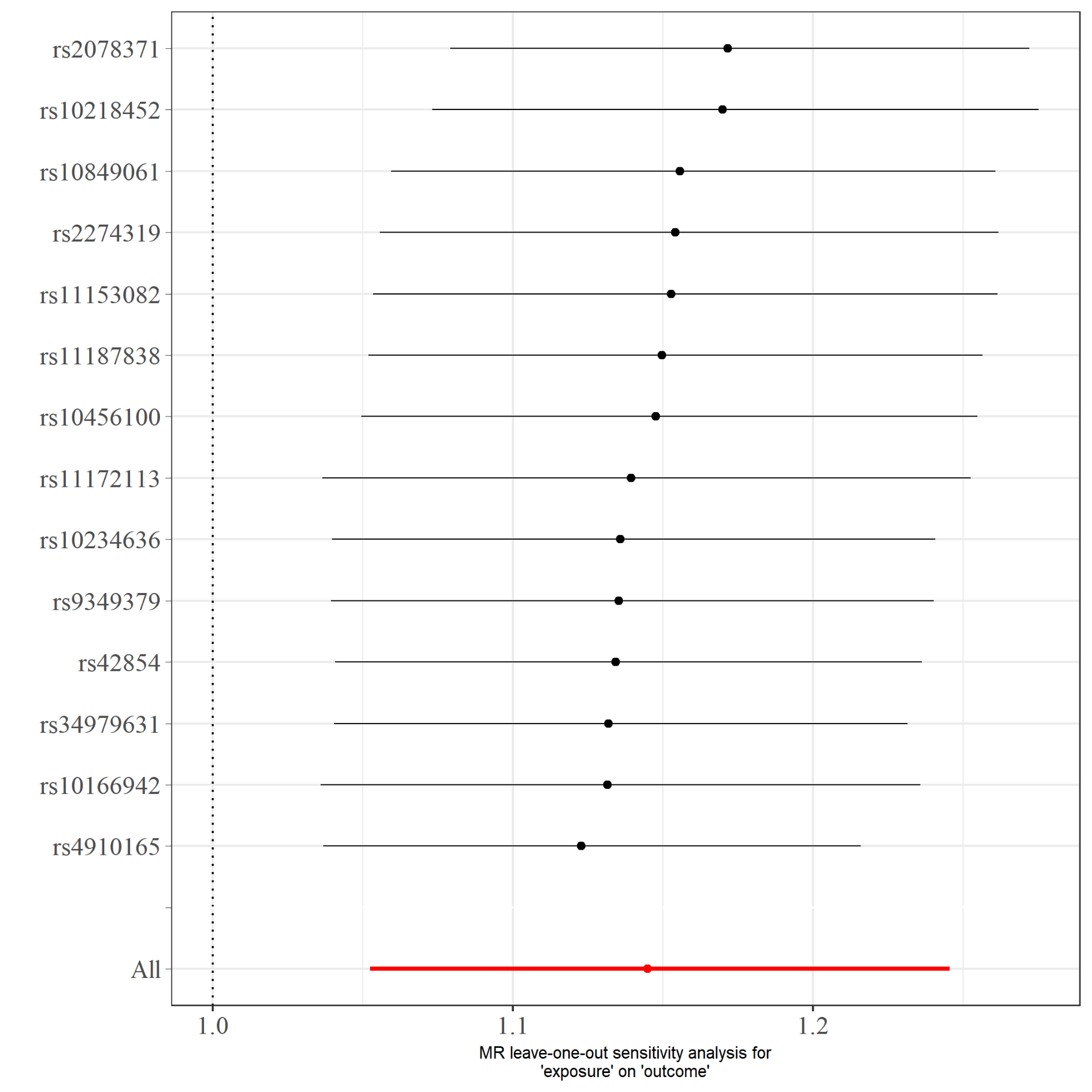
**

**Figure S7. MR effects of migraine on AD and longitudinal brain measures after removing potential pleiotropic IVs**

**
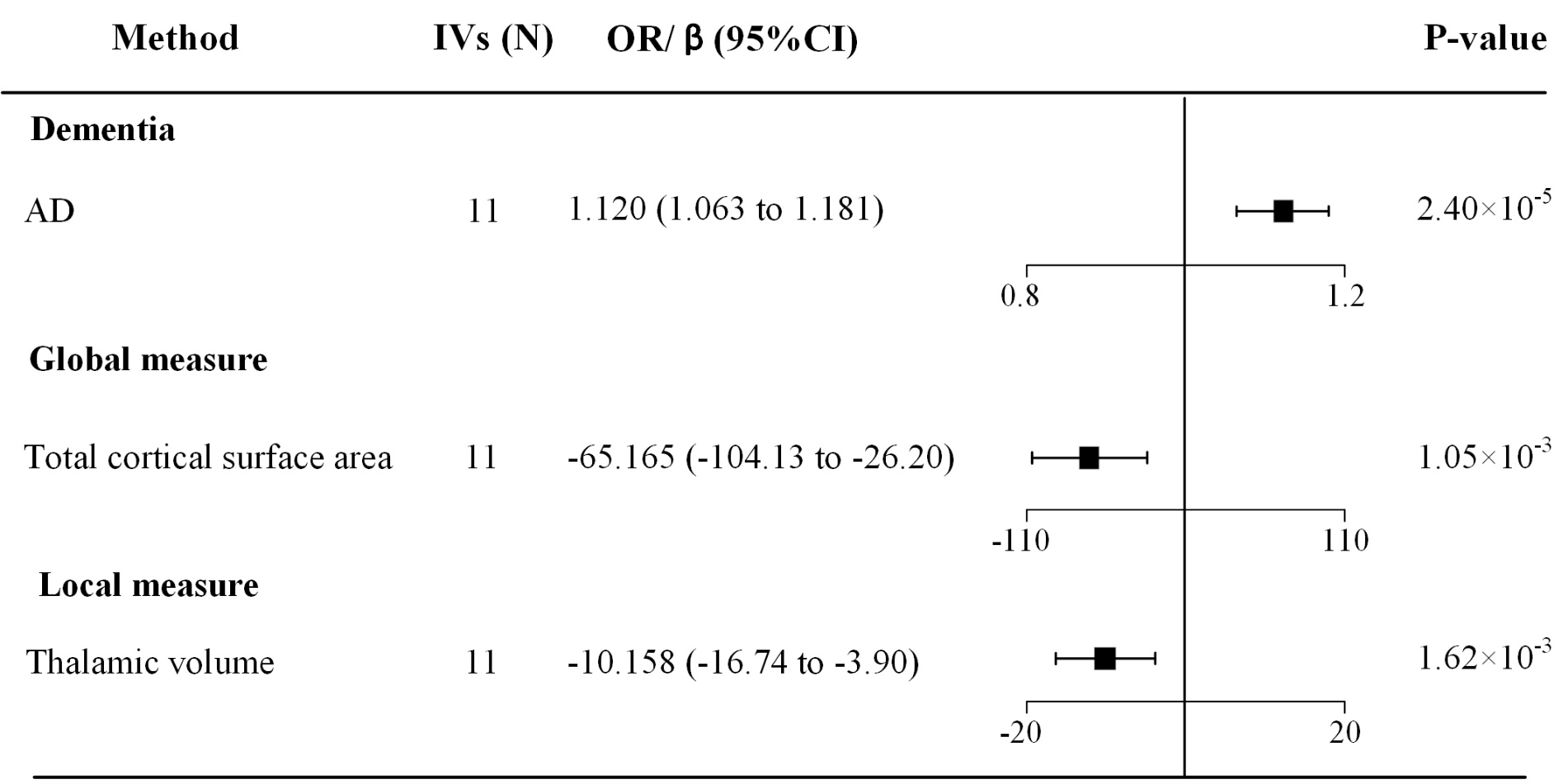
Abbreviations:** AD, Alzheimer's disease; IVs, instrumental variables; OR, odds ratio; CI, confidence interval.

**Figure S8. MR effects of MO on AD and longitudinal brain measures after removing potential pleiotropic IVs**

**
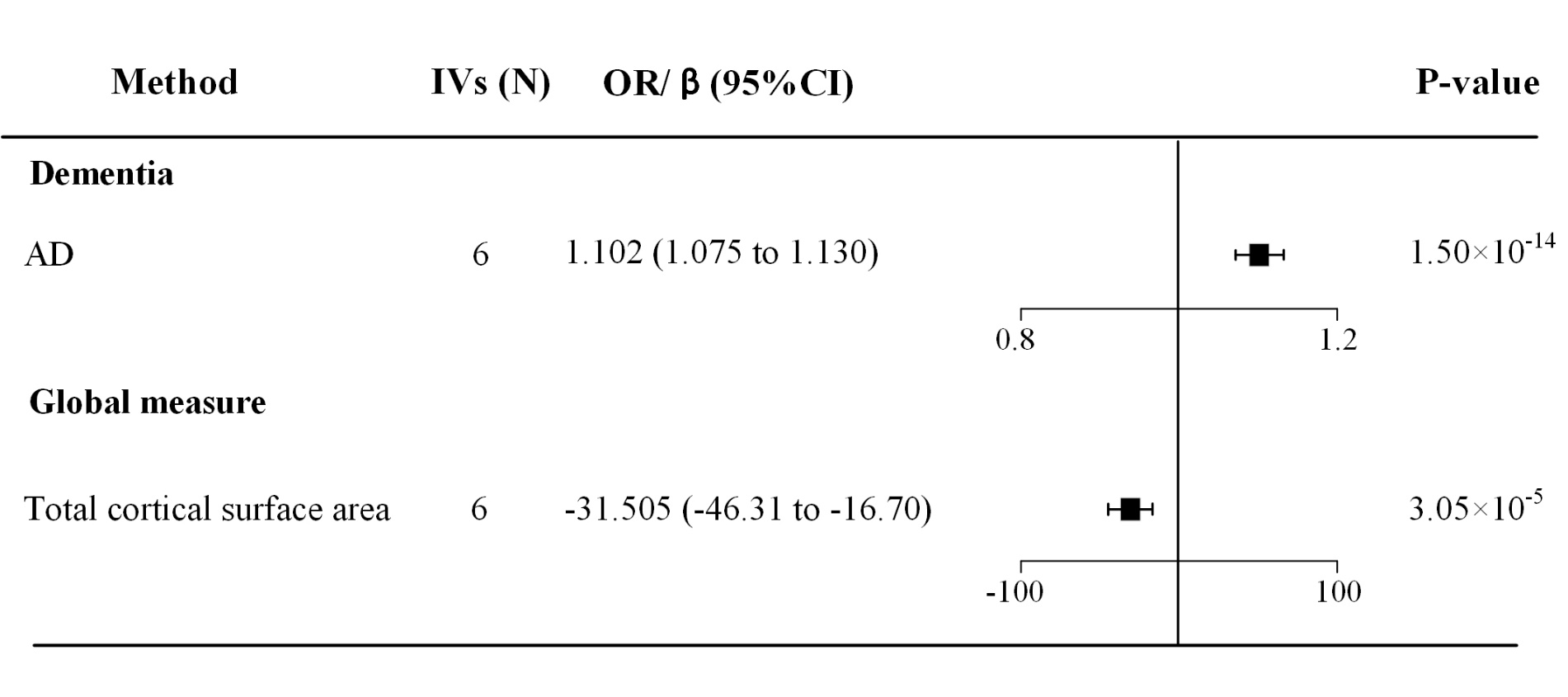
Abbreviations:** AD, Alzheimer's disease; IVs, instrumental variables; OR, odds ratio; CI, confidence interval.

**Figure S9. Supplementary MR estimates between migraine and AD**

**
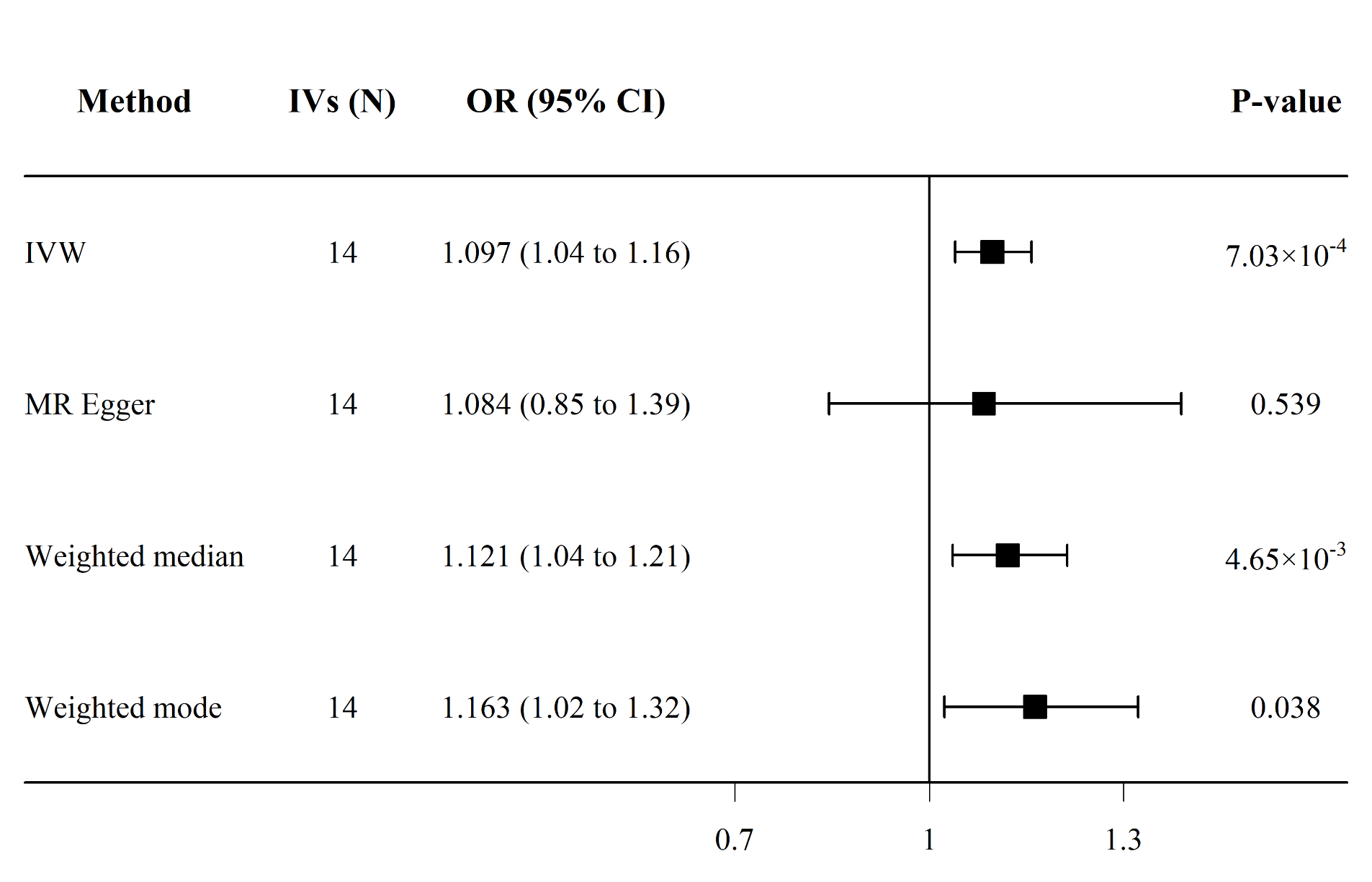
Abbreviations:** IVs, instrumental variables; OR, odds ratio; CI, confidence interval; IVW, multiplicative random-effects inverse-variance weighted.

**Figure S10. Supplementary MR estimates between migraine and total cortical surface area**
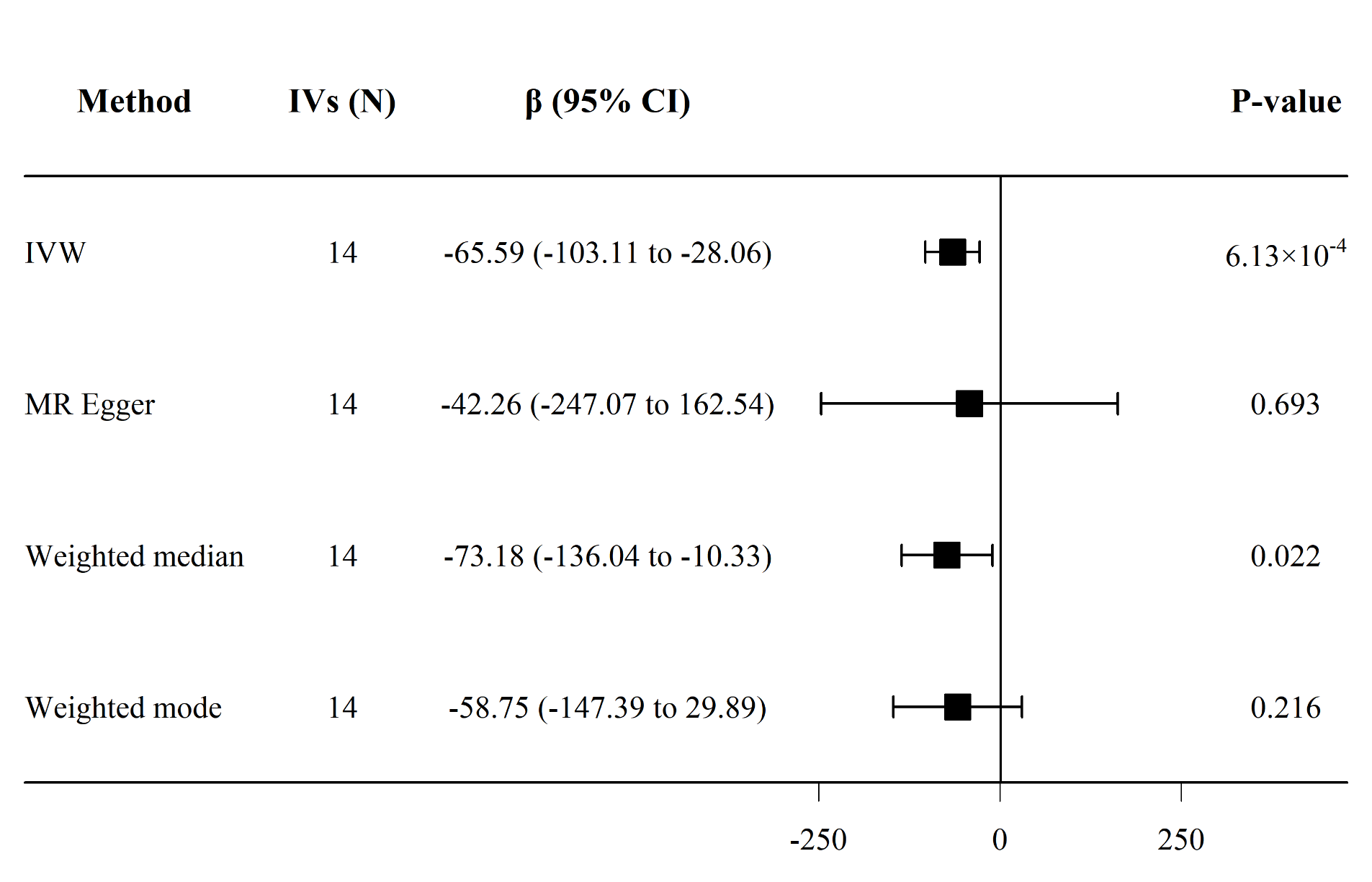
**Abbreviations:** IVs, instrumental variables; OR, odds ratio; CI, confidence interval; IVW, multiplicative random-effects inverse-variance weighted.

**Figure S11. Supplementary MR estimates between migraine and thalamic volume**


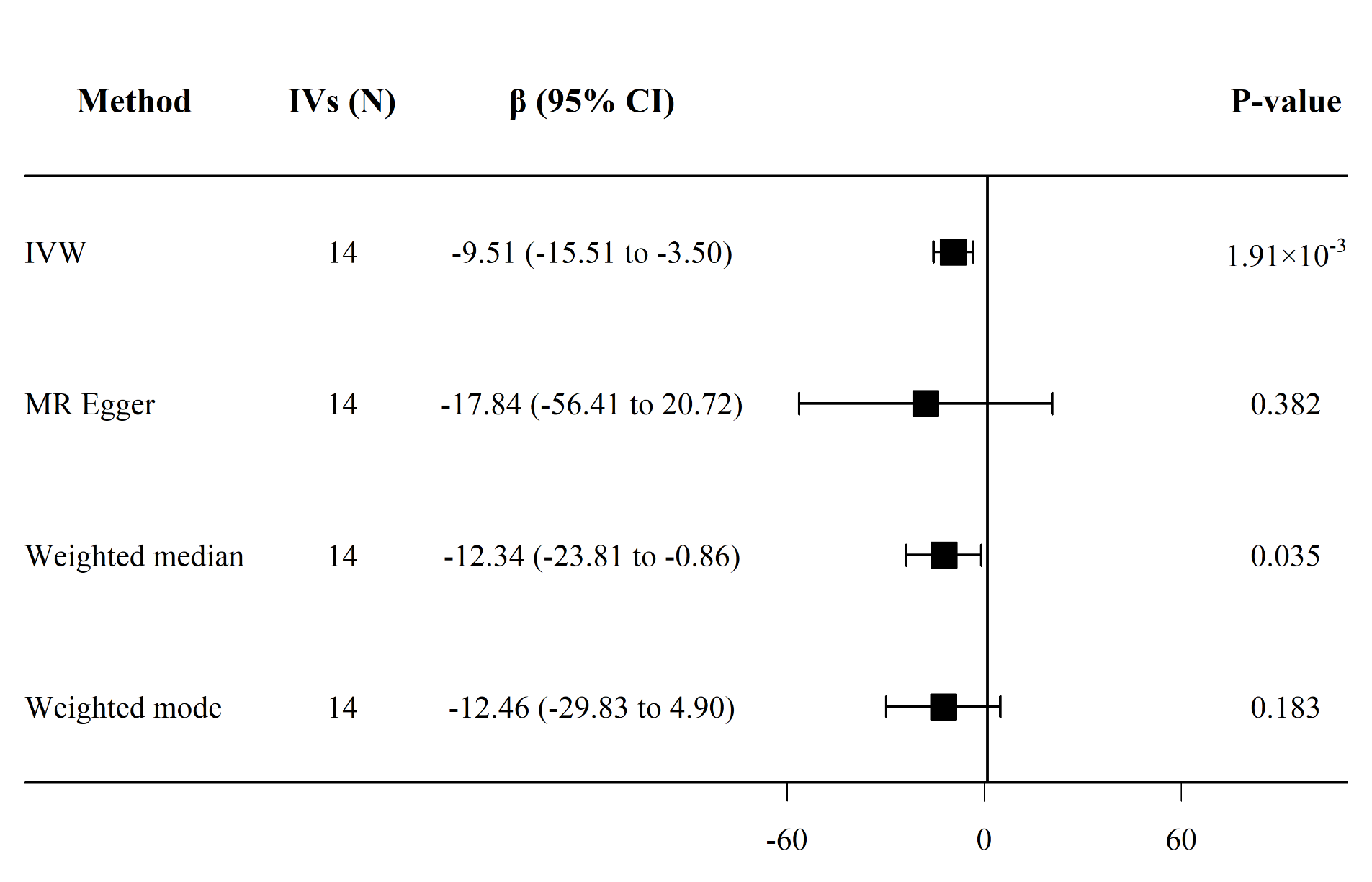
**Abbreviations:** IVs, instrumental variables; OR, odds ratio; CI, confidence interval; IVW, multiplicative random-effects inverse-variance weighted.

**Figure S12. Supplementary MR estimates between MO and AD**


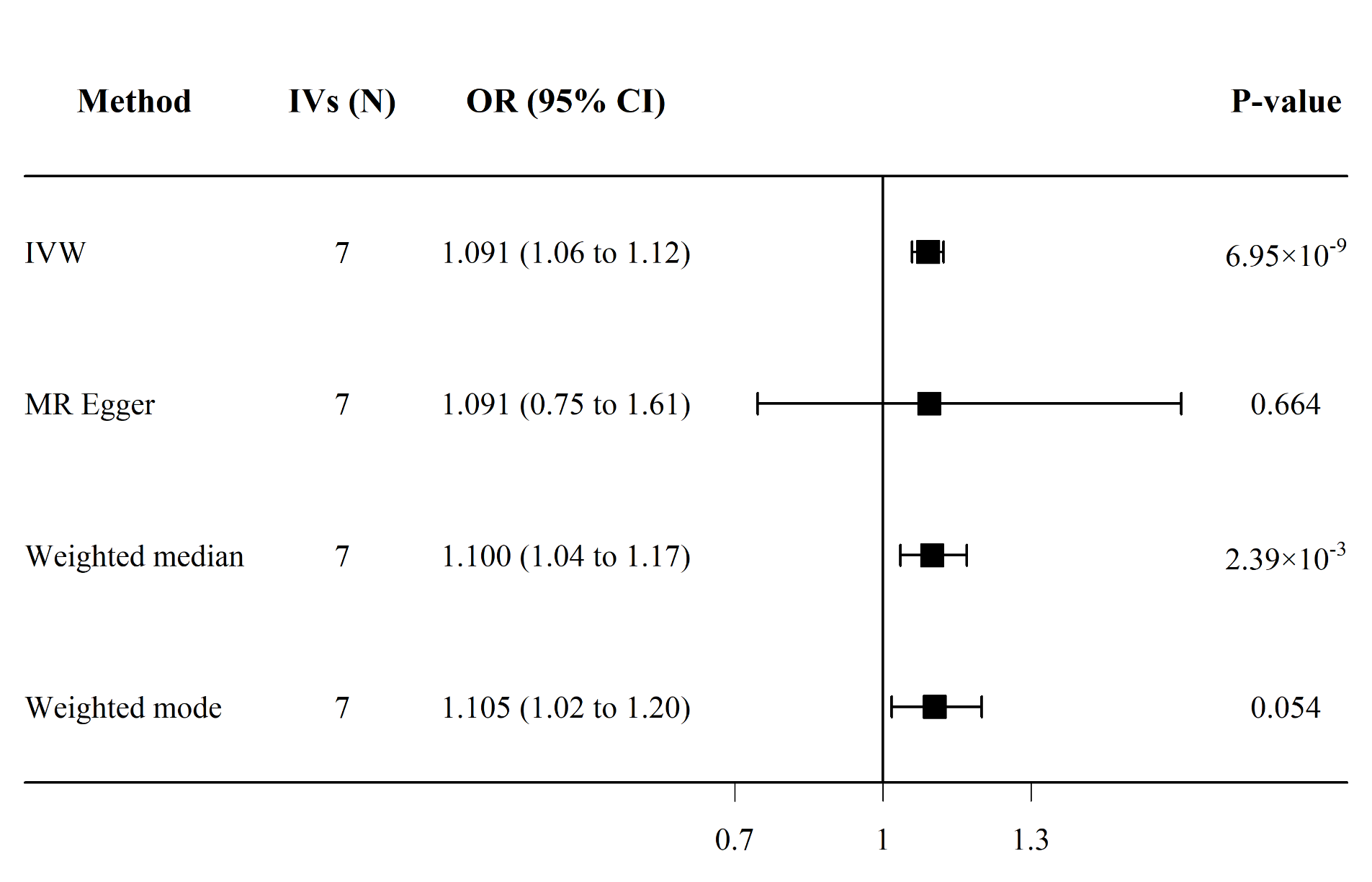
**Abbreviations:** IVs, instrumental variables; OR, odds ratio; CI, confidence interval; IVW, multiplicative random-effects inverse-variance weighted.

**Figure S13. Supplementary MR estimates between MO and total cortical surface area**


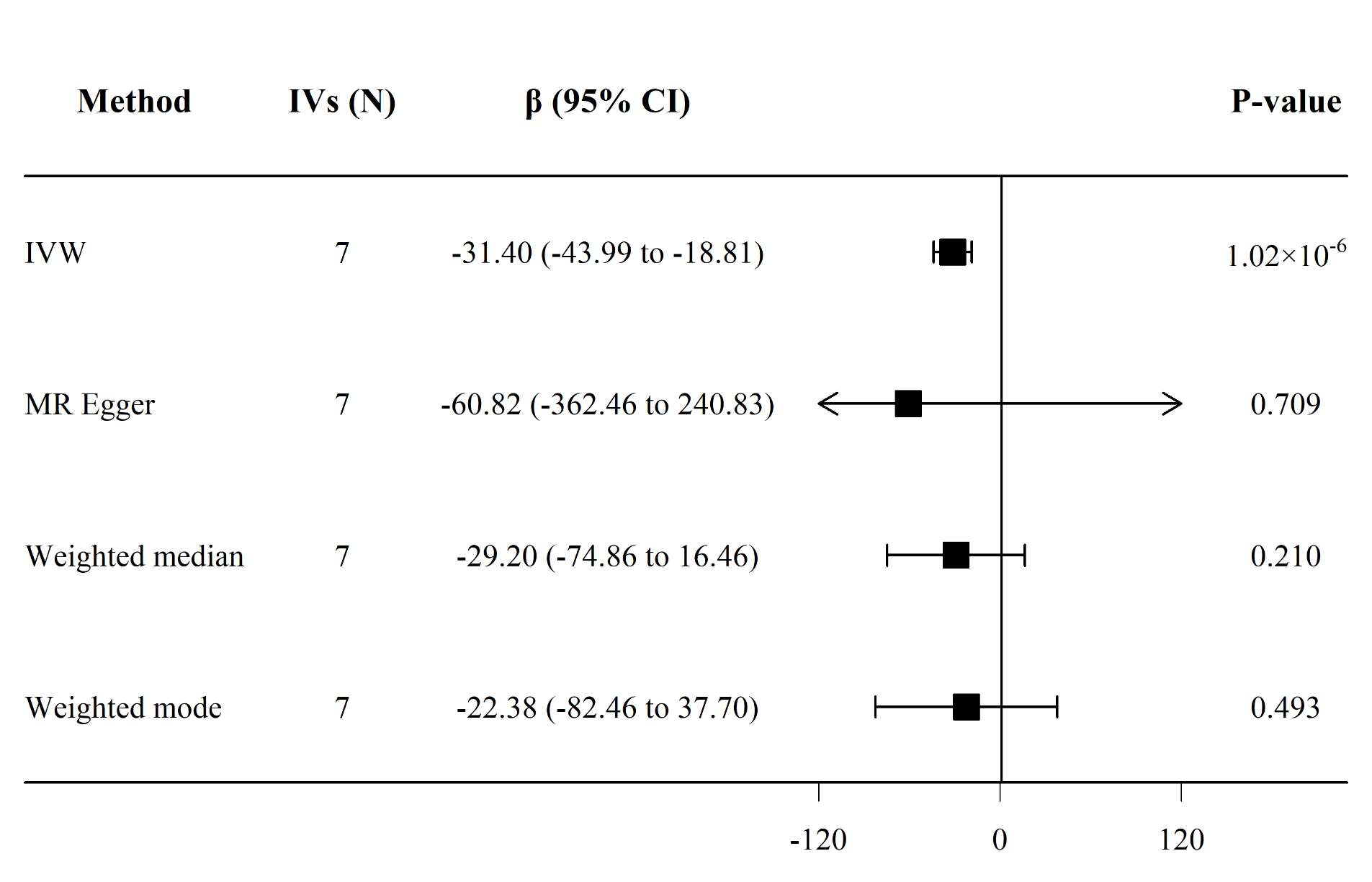
**Abbreviations:** IVs, instrumental variables; OR, odds ratio; CI, confidence interval; IVW, multiplicative random-effects inverse-variance weighted.

**Figure S14. Supplementary MR estimates between migraine and AD using the GWAS of AD in the FinnGen database**

**
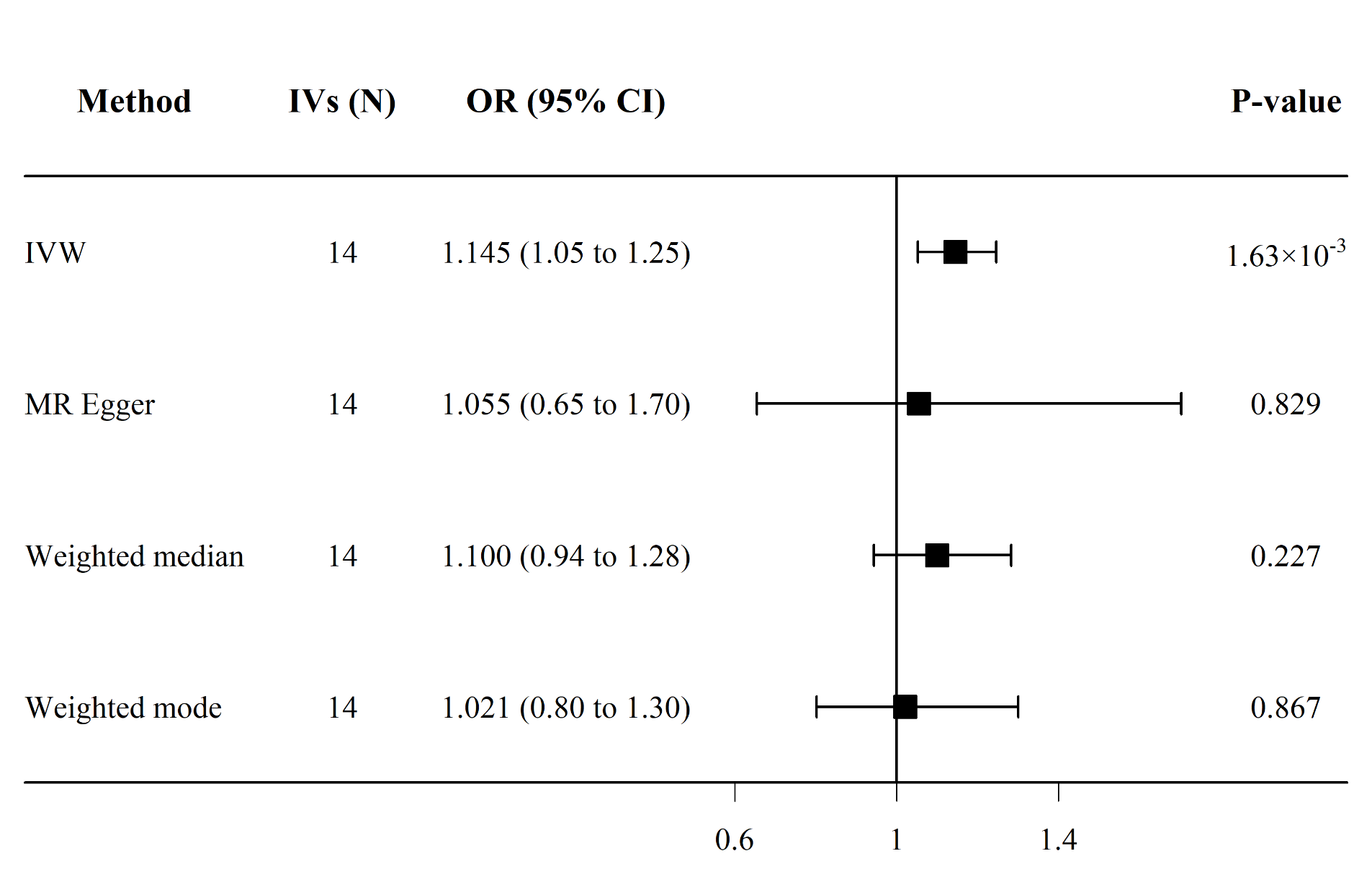
Abbreviations:** IVs, instrumental variables; OR, odds ratio; CI, confidence interval; IVW, multiplicative random-effects inverse-variance weighted.

**Figure S15. Reversed MR estimates between migraine and AD**

**
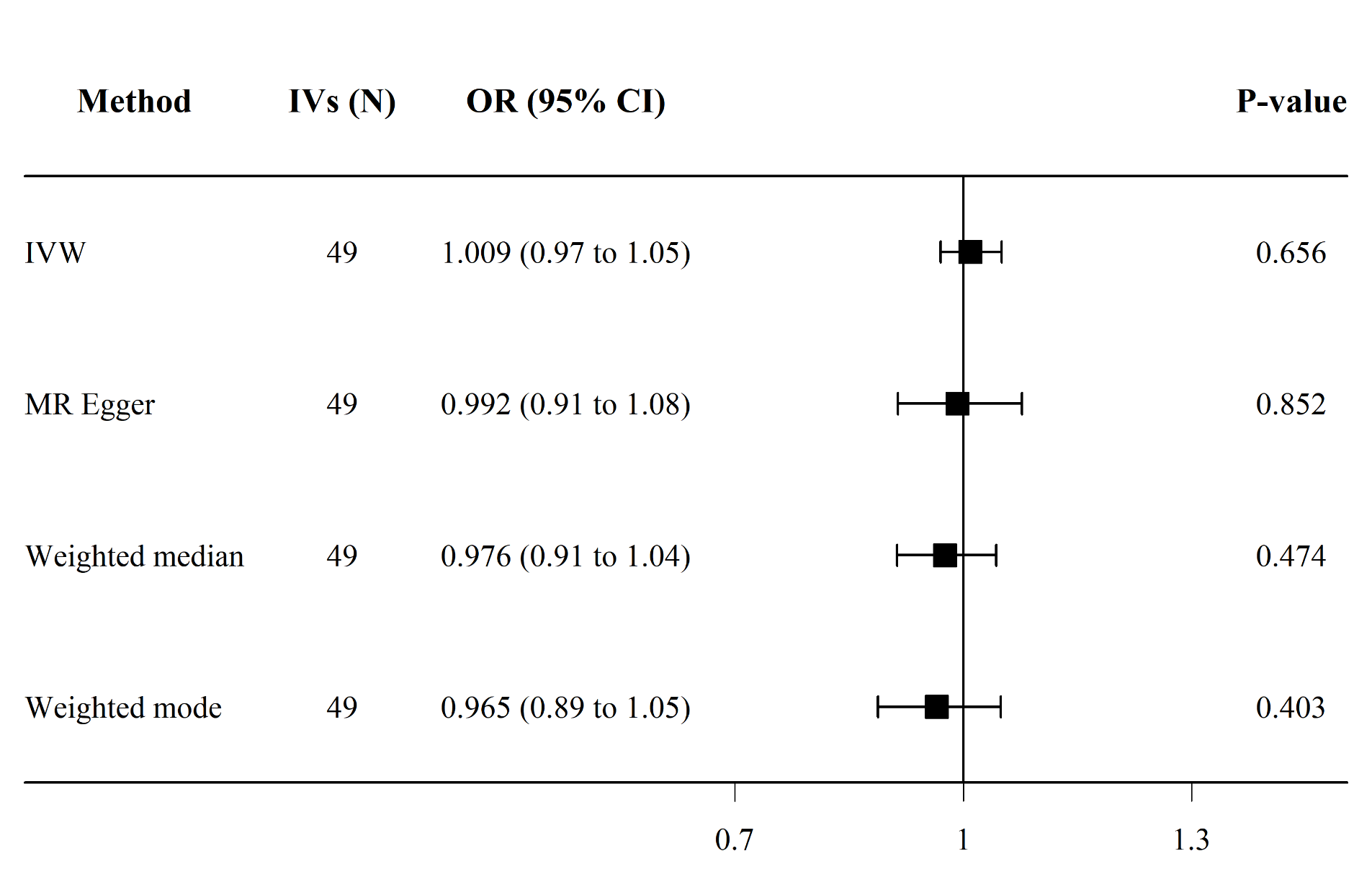
**

**Abbreviations:** IVs, instrumental variables; OR, odds ratio; CI, confidence interval; IVW, multiplicative random-effects inverse-variance weighted.

**Figure S16. MR effects of migraine on four longitudinal local brain measures not included in the primary analysis**


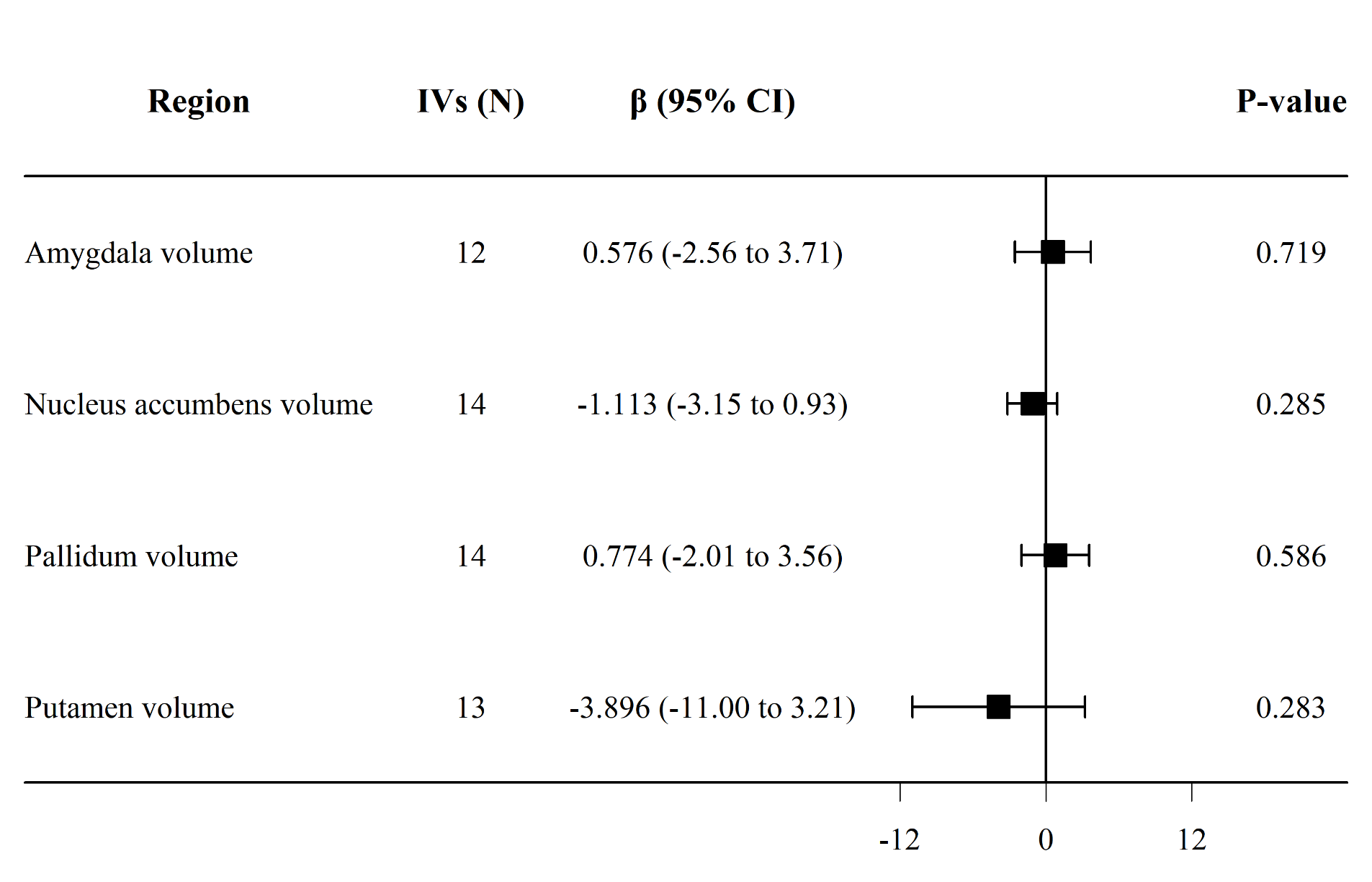


Abbreviations: IVs, instrumental variables; CI, confidence interval.
